# Effective production of kojic acid in engineered *Aspergillus niger*

**DOI:** 10.1186/s12934-023-02038-w

**Published:** 2023-02-27

**Authors:** Liu Wu, Licheng Zhang, Xiaojie Li, Ruitong Lv, Wei Cao, Weixia Gao, Jiao Liu, Zhoujie Xie, Hao Liu

**Affiliations:** 1 MOE Key Laboratory of Industrial Fermentation Microbiology, College of Biotechnology, Tianjin University of Science & Technology, Tianjin, 300457 China; 2 Tianjin Engineering Research Center of Microbial Metabolism and Fermentation Process Control, Tianjin University of Science & Technology, Tianjin, 300457 China; 3 National Technology Innovation Center of Synthetic Biology, 300308 Tianjin, People’s Republic of China

**Keywords:** Kojic acid, *Aspergillus niger*, Heterologous expression, Negative regulator

## Abstract

**Background:**

Kojic acid (KA) is a widely used compound in the cosmetic, medical, and food industries, and is typically produced by *Aspergillus oryzae*. To meet increasing market demand, it is important to optimize KA production through seeking alternatives that are more economic than current *A. oryzae*-based methods.

**Results:**

In this study, we achieved the first successful heterologous production of KA in *Aspergillus niger*, an industrially important fungus that does not naturally produce KA, through the expression of the *kojA* gene from *A. oryzae*. Using the resulting KA-producing *A. niger* strain as a platform, we identified four genes (*nrkA*, *nrkB*, *nrkC*, and *nrkD*) that negatively regulate KA production. Knocking down *nrkA* or deleting any of the other three genes resulted in a significant increase in KA production in shaking flask cultivation. The highest KA titer (25.71 g/L) was achieved in a pH controlled batch bioreactor using the *kojA* overexpression strain with a deletion of *nrkC*, which showed a 26.7% improvement compared to the KA titer (20.29 g/L) that was achieved in shaking flask cultivation.

**Conclusion:**

Our study demonstrates the potential of using *A. niger* as a platform for studying KA biosynthesis and regulation, and for the cost-effective production of KA in industrial strain development.

**Supplementary Information:**

The online version contains supplementary material available at 10.1186/s12934-023-02038-w.

## Background

**Fig. 1:**
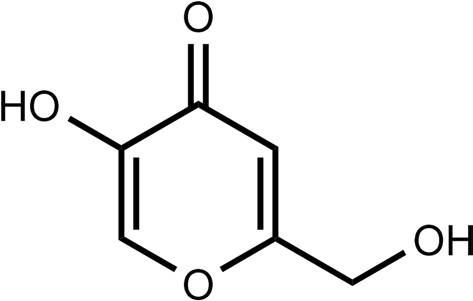
Structure of kojic acid

Kojic acid (KA, Fig. 1) is a secondary metabolite produced by a limited range of microorganisms, including *Aspergillus oryzae, Aspergillus flavus*, *Aspergillus tamari*, and several other species during the stationary phase of growth [1]. KA has been used as a food additive as an antioxidant, preservative, and flavor enhancer [2]. It has also been used as a skin lightening or bleaching agent in the cosmetics industry [1, 3]. In addition, KA is becoming an important starting molecule in medicinal chemistry research as KA and many KA derivatives have shown to exhibit anti-cancer, anti-inflammatory, insecticidal, antifungal, antibacterial, and antiviral properties [3–6].

Due to its wide range of commercial applications, the industrial production of KA has significantly increased. Chemical synthesis of KA was achieved in 1930 [7], but it has not been able to effectively meet the rising demand for the compound. The production of KA through aerobic fermentation of *Aspergillus* species is considered a safe and non-toxic technique. *A. oryzae*, the native KA producer, is currently used for industrial production of the compound [1].

KA biosynthesis in *A. oryzae* has been extensively studied using isotope tracer methods in the 1950s [8–12]. The results of these isotope labeling studies suggest that the direct conversion of glucose to KA without breaking the pyranose ring is a major pathway of KA formation in *A. oryzae*. The genes involved in KA biosynthesis in *A. oryzae* were first identified by Terabayashi et al. [13]. In their pioneering work, they revealed that two closely linked genes, AO090113000136 (FAD dependent oxidoreductase, named *kojA*) and AO090113000138 (a major facilitator superfamily (MFS) transporter, named *kojT*) in the genome of *A. oryzae* might be responsible for the production of KA [13]. KojA and KojT were proposed to be directly involved in the biosynthesis and secretion process, respectively. Further study indicated that a third gene located between *kojA* and *kojT,* AO090113000137 (Zn(II)2Cys6 transcription factor encoding gene, named *kojR*), is also involved in KA production through the regulation of the transcriptional expression of *kojA* and *kojT* [14]. In addition to the pathway-specific regulator *kojR*, KA biosynthetic genes could also be regulated by more global regulators, including the global transcriptional regulation gene *laeA* and the nitrate transporter-encoding gene *nrtA* [15, 16]. It is believed that the culture period-dependent production of KA is related to these global regulators. Studies also showed that KA production may be regulated by numerous other factors in *A. oryzae*, such as KpeA, Aokap1, Aokap2, Aokap4, and Aokap6 [17–21].

To date, *kojA* is the only enzyme-encoding gene confirmed to be involved in the biosynthesis of KA. However, the specific reaction catalyzed by KojA in the KA biosynthesis pathway remains unknown. It is also not clear if any other pathway-specific genes are required for the process. The lack of understanding of the KA biosynthesis pathway hinders efforts to genetically improve KA production. To gain a better understanding of KA biosynthesis and regulation, we decided to study it in the heterologous host of *A. niger*. *A. niger* was selected for several reasons: firstly, its conidia are uninucleate, in contrast to the multinucleate conidia of *A. oryzae* (which typically have two to four or more nuclei) [22], making genetic manipulation of *A. niger* easier compared to *A. oryzae*. Secondly, *A. niger* has a long history of safe use in the production of enzymes and organic acids, and has shown excellent performance in the production of organic acids such as citric acid and malic acid [23, 24]. This makes *A. niger* a good candidate for the development of an acidogenic chassis. In addition, *A. niger* grows on a wide range of substrates under various environmental conditions [25], which can be helpful in establishing a cost-effective fermentation process.

In this study, we achieved the heterologous production of KA in *A. niger* for the first time by reconstructing the biosynthetic pathway from *A. oryzae*. Starting from the KA-producing *A. niger*, we constructed mutant strains with knockout or knockdown of each of the homologs of genes in the predicted gene cluster for KA biosynthesis (ranging from AO090113000132 to AO090113000145 in *A. oryzae*). From this mutant library, we identified four genes that had negative regulatory functions in KA production. These findings demonstrate that *A. niger* is a useful platform for studying KA biosynthesis and regulation, and that *A. niger*-based cell factories have significant potential in creating industrial strains for cost-effective KA production.

## Results

### In silico comparison of the putative KA biosynthetic genes of *A. oryzae* to the *A. niger* strain ATCC 1015 genome

It has been reported that three genes, *AO090113000136* (*kojA*), *AO090113000137* (*kojR*) and *AO090113000138* (*kojT*), are involved in the biosynthesis of KA in *A. oryzae* RIB40 [13, 14]. These three closely linked genes are located in a gene cluster ranging from *AO090113000132* to *AO090113000145* [13]. Comparative genomics of *Aspergillus nidulans*, *Aspergillus fumigatus*, and *A. oryzae* also showed that this gene cluster is specific to *A. oryzae* [26]. Many secondary metabolism-related genes are often clustered in the genome [27], so the genes in the *A. oryzae*-specific gene cluster may have functions related to KA biosynthesis. However, the roles of most of the genes in the cluster in KA production, aside from *kojA*, *kojR*, and *kojT*, have not been well studied.

To determine if the homologs of the putative KA biosynthetic genes are present in *A.niger*, we performed a homology search using BLAST based on the *A. niger* genome sequence available from the NCBI. The alignment sequences with the most similarities were selected. As shown in Table 1, homologs for most of the genes in the putative KA biosynthetic gene cluster were found in the genome of *A. niger,* except for *kojA* and *AO090113000145*. All these genes have high sequence similarity with their homologs in *A. niger* (between 50 and 88%). It is worth noting that *AO090113000141* and *AO090113000142* match the same gene (*ASPNIDRAFT_209619*) in the genome of *A. niger*. *AO090113000141* and *AO090113000142* encode proteins with 243 and 187 amino acids, respectively, while their homolog (*ASPNIDRAFT_209619*) in *A. niger* encodes a protein with a length of 673 amino acids. Sequence alignment showed that the proteins encoded by *AO090113000141* and *AO090113000142* align well with the central and C-terminal parts of the protein encoded by *ASPNIDRAFT_209619*, respectively (Additional file 1: Fig. S1), indicating that there has been gene fusion/fission during the evolution of the corresponding proteins.

**Table 1:** Identifying homologs in *A. niger* ATCC 1015 for putative KA biosynthesis genes in *A. oryzae* RIB40A

Putative KA biosynthetic genes in *A. oryzae* (Locus tag/gene name)	Homologs in *A. niger* (Locus tag/gene name)	Putative function	Protein Identity /Similarity (%)
AO090113000132/-	ASPNIDRAFT_50239/-	DUF3759 domain-containing protein	57/67
AO090113000133/*Aokap6*	ASPNIDRAFT_171597/-	Unknown function	33/50
AO090113000134/-	ASPNIDRAFT_42619/-	DsbA-like oxidoreductase	41/57
AO090113000136/*kojA*	–	FAD oxidoreductase	–
AO090113000137/*kojR*	ASPNIDRAFT_189096/-	Transcription factor	63/78
AO090113000138/*kojT*	ASPNIDRAFT_43217/-	MFS transporter	78/87
AO090113000139/*Aokap4*	ASPNIDRAFT_53284/-	MFS transporter	78/88
AO090113000140/-	ASPNIDRAFT_56871/*nrkA*	Unknown function	71/82
AO090113000141/-	ASPNIDRAFT_209619/*nrkB*	Transcription factor	52 /64
AO090113000142/-	ASPNIDRAFT_209619/* nrkB*	Transcription factor	54/73
AO090113000143/-	ASPNIDRAFT_186610/*nrkC*	Unknown function	62/75
AO090113000144/-	ASPNIDRAFT_131173/*nrkD*	Sulfatase	71/82
AO090113000145/-	–	Unknown function	–

Therefore, for 11 of the 13 genes in the putative KA biosynthetic gene cluster, 10 homologs (corresponding to 10 genes scattered in different loci) were found in the genome of *A. niger* (Table 1). The *A. niger* genome does not have close homologs of either *kojA* or *AO090113000145*.

### Heterologous production of KA in *A. niger*

As analyzed above, *A. niger* lacks the homologs of *kojA* and *AO090113000145* among the genes in the putative KA biosynthetic cluster. Given that *kojA* is an enzyme-encoding gene that has been confirmed to be involved in KA biosynthesis, we focused on *kojA* for the reconstitution of the KA biosynthesis pathway in *A. niger*.

**Fig. 2:**
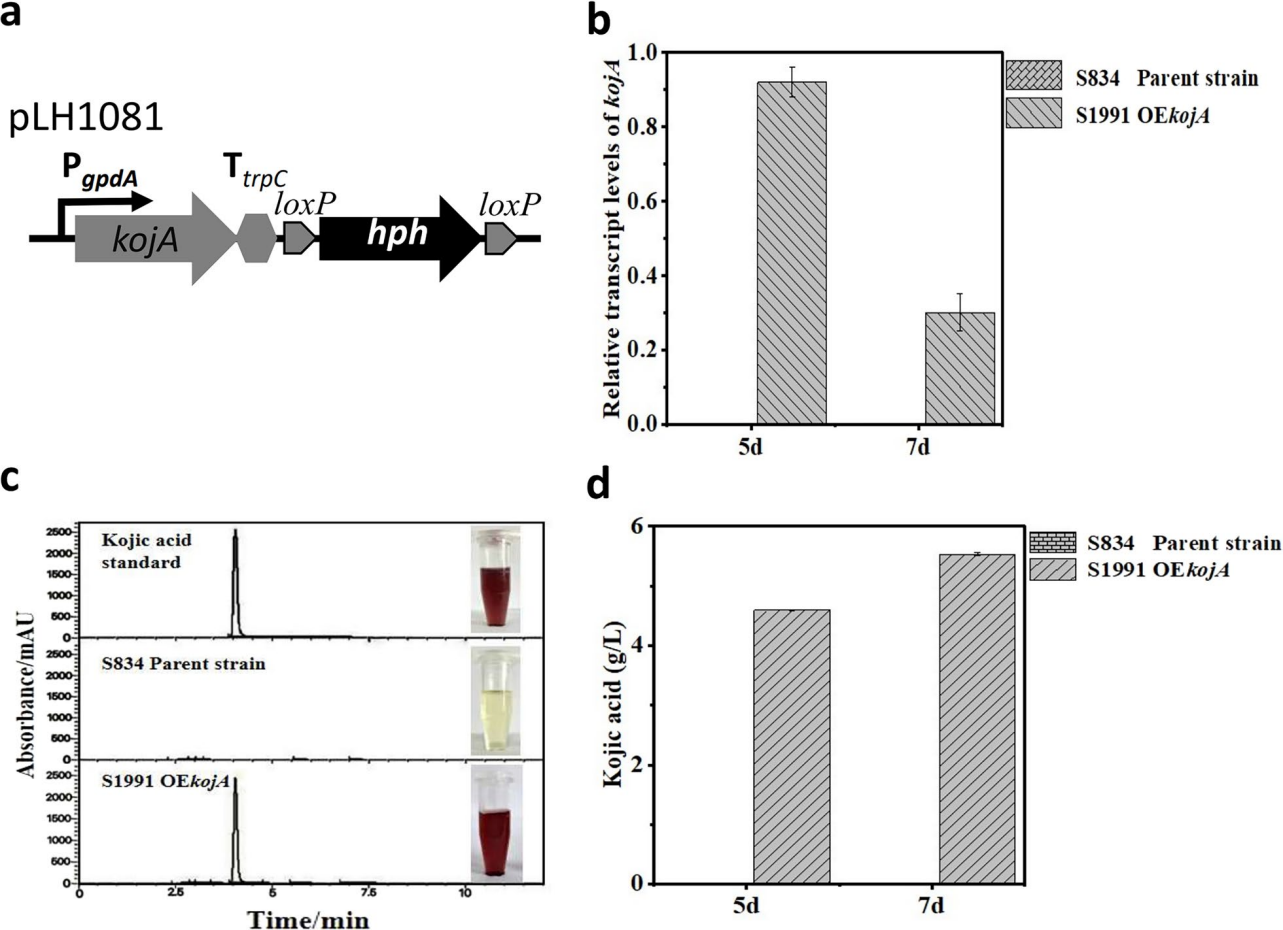
KA production in *A. niger* cell factory overexpressing *kojA* from *A. oryzae*. **a** Illustration of the genetic organization for the *kojA* overexpression cassette in pLH1081. **b** Expression levels of *kojA* in the parent strain S834 and *kojA* overexpression strain S1991 respectively. The transcriptional levels in each strain at 5 d and 7d after inoculation are indicated. All experiments measuring transcription via qRT-PCR were normalized using the actin gene as a housekeeping control. **c** KA can form a red chelated compound with ferric ions. Color reaction of 5 day culture of S1991 with ferric ions indicates the kojic acid secretion by strain S1991. Similar results were obtained by HPLC using the commercial KA as standard. **d** The amount of KA produced by *A. niger* S1991 in shake flasks for 5 days and 7 days was determined by HPLC. Commercial kojic acid was used as a standard

The expression of *kojA* in *A. oryzae* is dependent on the growth phase and culture conditions [13]. Its expression is also regulated by the pathway-specific regulator KojR [14], the global regulator LaeA [15], and several other regulators such as NrtA, KpeA, Aokap1, etc.[16–18]. To ensure its expression in *A. niger*, the *kojA* gene was placed under the control of the promoter of glyceraldehyde-3-phosphate dehydrogenase (P*gpdA*), a strong and constitutive endogenous promoter in *A. niger* [28], to create the expression cassette for *kojA* (Fig. 2a). Citric acid and oxalic acid are two major organic acids produced by *A. niger* ATCC 1015. To redirect glucose metabolic flux towards KA in the engineered *A. niger*, we used the double deletion strain of *cexA* and o*ahA* (*A. niger* S834), an *A. niger* ATCC 1015 derivative that is unable to produce citric acid and oxalic acid [29], as the host strain for the reconstitution of the KA biosynthesis pathway. The *kojA* overexpression cassette was integrated into the genome of *A. niger* S834 through *Agrobacterium*-mediated transformation (AMT) to obtain the *kojA* overexpression strain *A. niger* S1991 (OE*kojA*). The successful integration of the expression cassette was verified by PCR (Additional file 1: Fig. S2). qRT-PCR was performed to show the high expression of the introduced *kojA* gene in *A. niger* S1991 (Fig. 2b).

*A. niger* S1991 (OE*kojA*) was cultivated in the KA production medium, and the formation of KA was monitored in the supernatant using a colorimetric method [30]. The parental strain *A. niger* S834 was cultivated under the same conditions as a negative control. As shown in Fig. 2c, a red color was produced in the cultivation medium of *A. niger* S1991 after fermentation for 5 days when the colorimetric method was used to detect KA, while no color reaction was observed in the cultivation medium of strain *A. niger* S834, indicating the successful production of KA in strain *A. niger* S1991. HPLC analysis further confirmed the production of KA in the culture of *A. niger* S1991 (Fig. 2c). The yield of KA at 7 days of culture reached up to 5.53 g/L in strain S1991. No KA production was detected in the parental strain S834 (Fig. 2d). This demonstrates that the introduction of a single gene, *kojA*, is sufficient for KA production in *A. niger*. Considering that *AO090113000145* and its homologs were not included in the engineered KA producing *A. niger* S1991, it can be concluded that *AO090113000145* is dispensable for KA biosynthesis in *A. niger*.

### Roles of homologues of the putative KA biosynthesis genes in the production of KA in *A. niger*

As previously mentioned, 10 homologs corresponding to the 11 putative KA biosynthetic genes from *A. oryzae* were found in the genome of *A. niger* (Table 1). To determine the roles of these homologs in the production of KA, we attempted to construct disruption mutants for each of them in the genetic background of the KA-producing *A. niger* strain constructed above. To do this, we first eliminated the hygromycin resistance marker (*hph*) in *A. niger* S1991 using the Cre-*lox*P system [31], so that the selection marker for hygromycin could be used in the subsequent round of transformation. The successful excision of the *hph* gene, which confers resistance to hygromycin, was confirmed by PCR (Additional file 1: Fig. S3), and the resulting marker-less strain was designated as *A. niger* S2132. Starting with *A. niger* S2132, gene deletion experiments were performed for all 10 homologs separately using the *hph* gene replacement through homologous recombination. For 8 of them (*ASPNIDRAFT_50239*, *ASPNIDRAFT_171597*, *ASPNIDRAFT_189096*, *ASPNIDRAFT_43217*, *ASPNIDRAFT_53284*, *ASPNIDRAFT_209619*, *ASPNIDRAFT_186610*, *ASPNIDRAFT_131173*), individual deletion mutants were successfully obtained. The correct gene replacement in each deletion mutant was confirmed by PCR (See Additional file 1: Fig. S4), and the confirmed deletion mutants were designated as *A. niger* S2624 (∆*ASPNIDRAFT_50239*), S2922 (∆*ASPNIDRAFT_171597*), S2924(∆*ASPNIDRAFT_189096*), S2929(∆*ASPNIDRAFT_43217*), S2626 (∆*ASPNIDRAFT_53284*), S2430 (∆*ASPNIDRAFT_209619*), S2435 (∆*ASPNIDRAFT_186610*), S2437 (∆*ASPNIDRAFT_131173*) respectively.

**Fig. 3:**
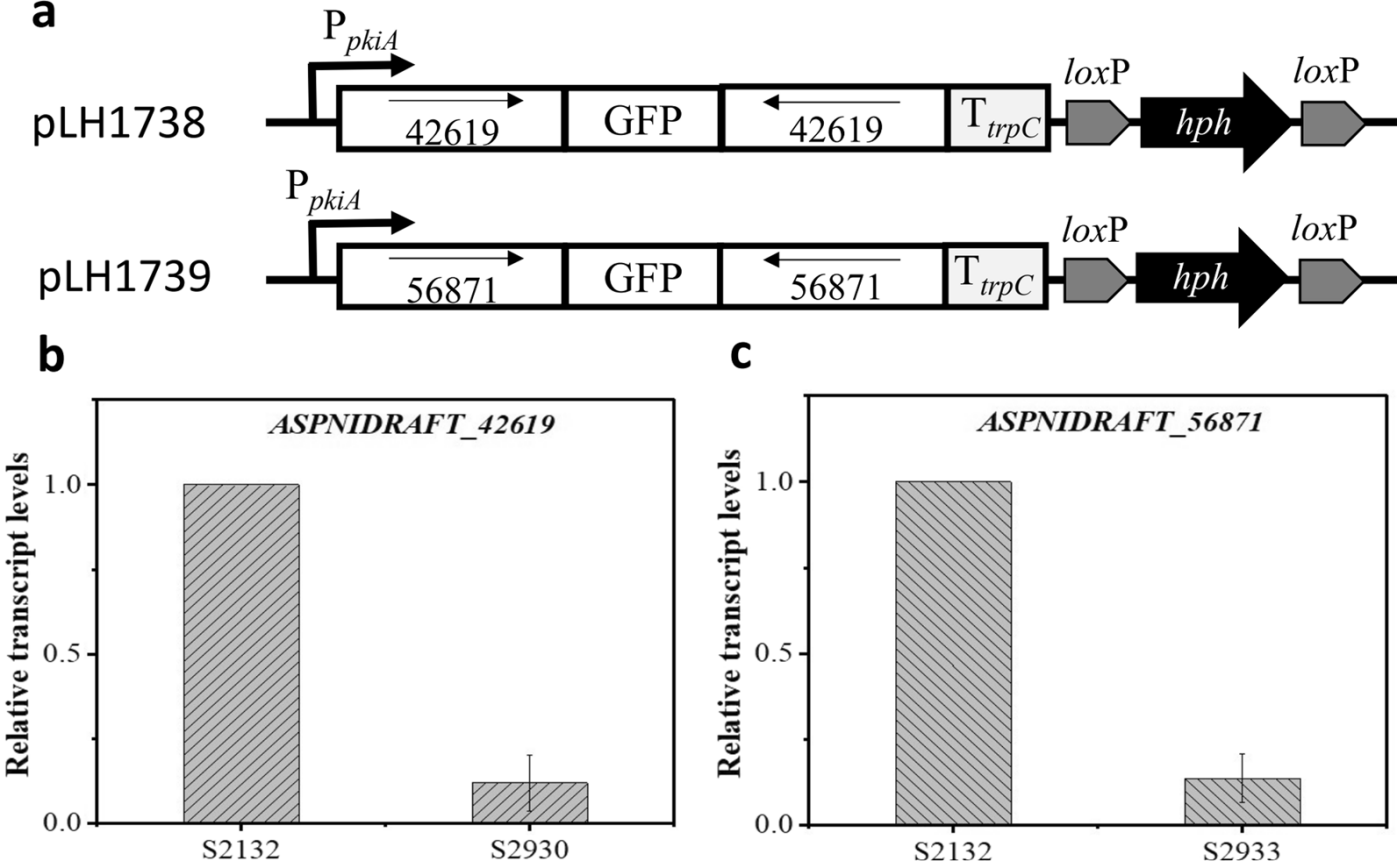
Construction of *A. niger* strains with RNA interference targeting *ASPNIDRAFT_42619* and *ASPNIDRAFT_56871* respectively. **a** Illustration of RNAi cassettes designed with inverted repeats of 500 bp of coding sequence of the gene of interest separated by a spacer segment of GFP sequence. pLH1738 was used to interfere with *ASPNIDRAFT_42619* expression, pLH1739 to interfere with *ASPNIDRAFT_56871* expression. **b** qRT-PCR analysis of target gene expression for the parent strain S2132 and the RNAi strains. the expression of *ASPNIDRAFT_42619* and *ASPNIDRAFT*_*56871* was interfered with in S2930 and S2933 respectively. Results were first standardized against actin, with S2132 expression set arbitrarily to 1

However, multiple attempts to obtain deletion mutants for *ASPNIDRAFT_42619* and *ASPNIDRAFT_56871* were unsuccessful, suggesting that both genes may be essential for the survival of *A. niger*. To overcome the difficulty of generating gene knockout strains for both genes, we used RNA interference (RNAi) technology to repress the expression of *ASPNIDRAFT_42619* and *ASPNIDRAFT_56871*, respectively. To do this, we used an RNAi initiated by a hairpin construct, where duplicate sequences of 500 bp of target gene were cloned as inverted repeats separated by a 101-bp spacer of green fluorescent protein (GFP) encoding sequence, as previously described in *Cryptococcus neoformans* [32]. To control the expression of the interfering RNA, we used the promoter of pyruvate kinase A gene (P*pkiA*) [28], which is a strong constitutive promoter used in *A. niger.* RNAi cassettes targeting *ASPNIDRAFT_42619* and *ASPNIDRAFT_56871* were constructed (Fig. 3a), and were introduced into *A. niger* S2132 through AMT. The correct insertion of the RNAi cassettes was confirmed by PCR and the confirmed RNAi constructs were designated as *A. niger* S2930 (RNAi-*ASPNIDRAFT_42619*) and *A. niger* S2933 (RNAi-*ASPNIDRAFT_56871*) respectively. qRT-PCR were conducted to measure the expression levels of *ASPNIDRAFT_42619* and *ASPNIDRAFT_56871* in *A. niger* S2930 and *A. niger* S2933, respectively. The results showed that the expression level of *ASPNIDRAFT_42619* in *A. niger* S2930 was 12% of that in the control strain *A. niger* S2132 (Fig. 3b), and the expression level of *ASPNIDRAFT_56871* in *A. niger* S2933 was 14% of that in the control strain (Fig. 3c). These results suggest that the expression of *ASPNIDRAFT_42619* and *ASPNIDRAFT_56871* was significantly suppressed in the corresponding RNAi strains.

**Fig. 4:**
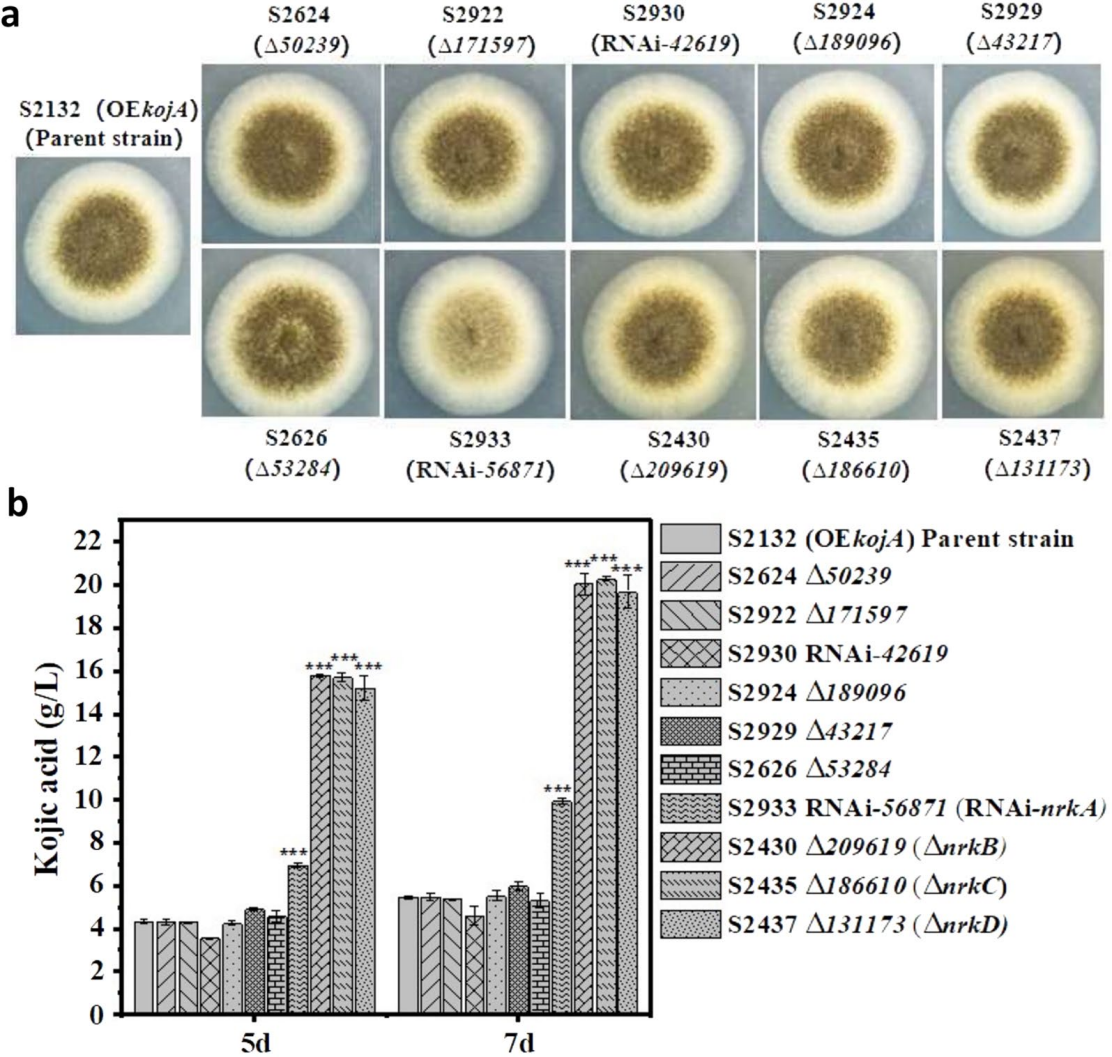
Screening of genes related with KA biosynthesis using kojic acid producing *A. niger* as a platform. **a** Colony Phenotype of 10 mutant strains grown on PDA for 4 days. The strains are listed as follows: the marker-less *kojA* overexpression strain S2132 (OE*kojA*) used as the parent strain, ASPNIDRAFT_50239 deletion mutant S2624 (Δ50239), ASPNIDRAFT_171597 deletion mutant S2922 (Δ171597), RNAi strain targeting ASPNIDRAFT_42619 S2930 (RNAi-42619), ASPNIDRAFT_189096 deletion mutant S2924 (Δ189096), ASPNIDRAFT_43217 deletion mutant S2929 (Δ43217), ASPNIDRAFT_53284 deletion mutant S2626 (Δ53284), RNAi strain targeting ASPNIDRAFT_56871 S2933 (RNAi-56871), ASPNIDRAFT_209619 deletion mutant S2430 (Δ209619), ASPNIDRAFT_186610 deletion mutant S2435 (Δ186610), ASPNIDRAFT_131173 deletion mutant S2437 (Δ131173). **b** KA production by the parent strain S2132 and the 10 derivative mutant strains. The strains are listed as described above. The titers of KA produced by *A. niger* S2132 and 10 derivative mutants in shake flask cultivations for 5 d and 7 d were shown

Out of the 10 constructions, including 8 gene deletion mutants and 2 RNAi strains, 9 of them had similar colony morphologies on PDA plate as the parent strain *A. niger* S2132. However, the RNAi strain *A. niger* S2933 (RNAi-*ASPNIDRAFT_56871*) showed a severe reduction in conidiation phenotype (Fig. 4a), indicating that *ASPNIDRAFT_56871* may play a crucial role in the morphological development of *A. niger*.

These 10 constructions were also cultivated in the KA production medium at 28 °C for 7 days, and the production of KA was monitored in the supernatant. The parental strain *A. niger* S2132 was grown under the same conditions as a control. As shown in Fig. 4b, the production of KA was significantly increased in strains S2933 (RNAi-*ASPNIDRAFT_56871*), S2430 (∆*ASPNIDRAFT_209619*), S2435 (∆*ASPNIDRAFT_186610*) and S2437 (∆*ASPNIDRAFT_131173*). These strains produced 1.82-fold (9.95 g/L), 3.67-fold (20.05 g/L), 3.71-fold (20.29 g/L), and 3.60-fold (19.70 g/L) titer that was achieved in the control strain S2132 (5.47 g/L), respectively, after fermentation for 7 days. This suggests that these four genes function as negative regulator in KA production. Therefore, the four genes of *ASPNIDRAFT_56871*, *ASPNIDRAFT_209619*, *ASPNIDRAFT_186610* and *ASPNIDRAFT_131173* were designated as *nrkA* (negative regulator of KA production A), *nrkB*, *nrkC* and *nrkD* respectively in this study.

The production of KA in the remaining 6 strains did not show a statistically significant difference compared to the parental strain (Fig. 4b). This indicate that the corresponding 6 genes may not be involved in KA biosynthesis.

### Effects of multiple gene disruption (silencing) on KA production in *A. niger*

As demonstrated above, four genes (*nrkA*, *nrkB*, *nrkC* and *nrkD*) that function as negative regulators of KA production were identified in *A. niger*. We then sought to determine whether the combined disruption of these negative regulator encoding genes could further increase KA production. To do this, we first eliminated the *hph* gene from the high-yielding strain S2435 (Δ*nrkC*) using the Cre-loxP system. The resulting marker-less strain *A. niger* S2743 (Δ*nrkC*) was used as the starting strain for the next round of transformation. When we attempted to delete the remaining three negative regulator encoding genes from strain S2743 through homologous recombination, only *nrkD* was successfully deleted. The resulting strain was designated as *A. niger* S2684 (Δ*nrkC*, Δ*nrkD*). The failure to delete *nrkA* in strain S2743 (Δ*nrkC*) is consistent with our previous results from the single gene deletion study in *A. niger* S2132 (OE*kojA*). However, in contrast to our successful deletion of *nrkB* in *A. niger* S2132 (OE*kojA*), the failure to delete *nrkB* in S2743 (Δ*nrkC*) suggests that *nrkB* and *nrkC* may have redundant functions in an essential cellular physiological process. We then used RNAi technology to knockdown the expression of *nrkA* and *nrkB* in the genetic background of *nrkC* and *nrkD* double deletio*n*. Starting with *A. niger* S2684, and using the Cre-*lox*P system to efficiently recycle selection marker (*hph*), we performed two rounds of RNAi cassette transformation to obtain *A. niger* S3058 (RNAi-*nrkA*, Δ*nrkC*, Δ*nrkD*) and *A. niger* S3119 (RNAi-*nrkA*, RNAi-*nrkB*, Δ*nrkC*, Δ*nrkD*) respectively. The construction details of plasmids and strains are described in the Method part.

**Fig. 5:**
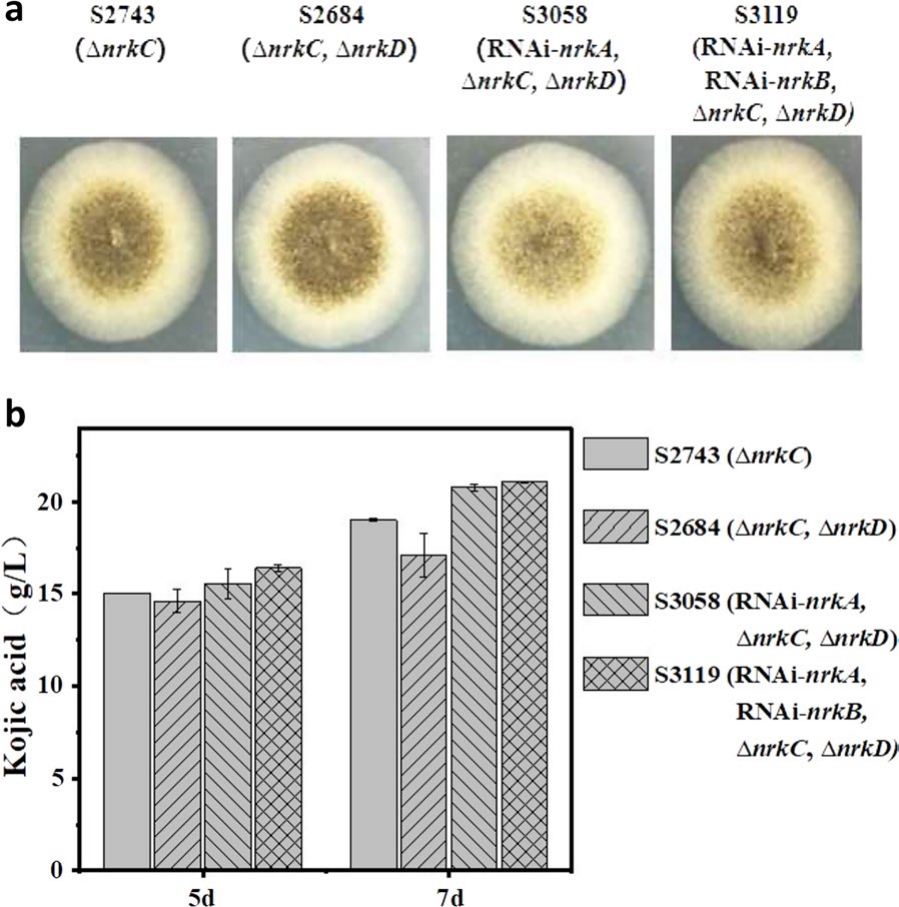
The effects of combined mutation of four negative regulator encoding genes on the kojic acid production. **a.** Colony Phenotype of four mutant strains grown on PDA for 3 days. The strains are listed as follows: the marker-less *nrkC* deletion mutant: S2743 (Δ*nrkC*), *nrkC* and *nrkD* double deletion mutant: S2683 (Δ*nrkC*, Δ*nrkD*), *nrkC* and *nrkD* double deletion mutant with RNAi targeting *nrkA*: S3058 (RNAi-*nrkA*, Δ*nrkC*, Δ*nrkD*), *nrkC* and *nrkD* double deletion mutant with RNAi targeting *nrkA* and *nrkB*: S3119 (RNAi-*nrkA*, RNAi-*nrkB*, Δ*nrkC*, Δ*nrkD*). All the four strains have the same genetic background of *kojA* overexpression except the genetic difference mentioned above. **b** KA production by the four engineered strains. The strains are listed as described above. The titers of KA produced by the four engineered strains in shake flask cultivations for 5 days and 7 days were shown

*A. niger* S2743 (Δ*nrkC*), *A. niger* S2684(Δ*nrkC*, Δ*nrkD*), *A. niger* S3058 (RNAi-*nrkA*, Δ*nrkC*, Δ*nrkD*) and *A. niger* S3119 (RNAi-*nrkA*, RNAi-*nrkB*, Δ*nrkC*, Δ*nrkD*) were inoculated in PDA, and the colony phenotype was compared. As shown in Fig. 5a, both S3058 and S3119 displayed severely reduced conidiation phenotype, which is similar with that of S2933 (RNAi-*nrkA*). The expression of *nrkA* is downregulated in all three strains by RNAi. The results further support the putative function of *nrkA* involved in the morphological development of *A. niger*. The four strains were cultivated in the KA production medium for 7 days, and the production of KA was monitored in the culture supernatant using HPLC. As shown in Fig. 5b, KA production did not significantly differ among them, indicating that multiple gene knockout (knockdown) of the four negative regulator encoding genes can not further increase KA production.

### KA production in pH controlled batch cultures

**Fig. 6:**
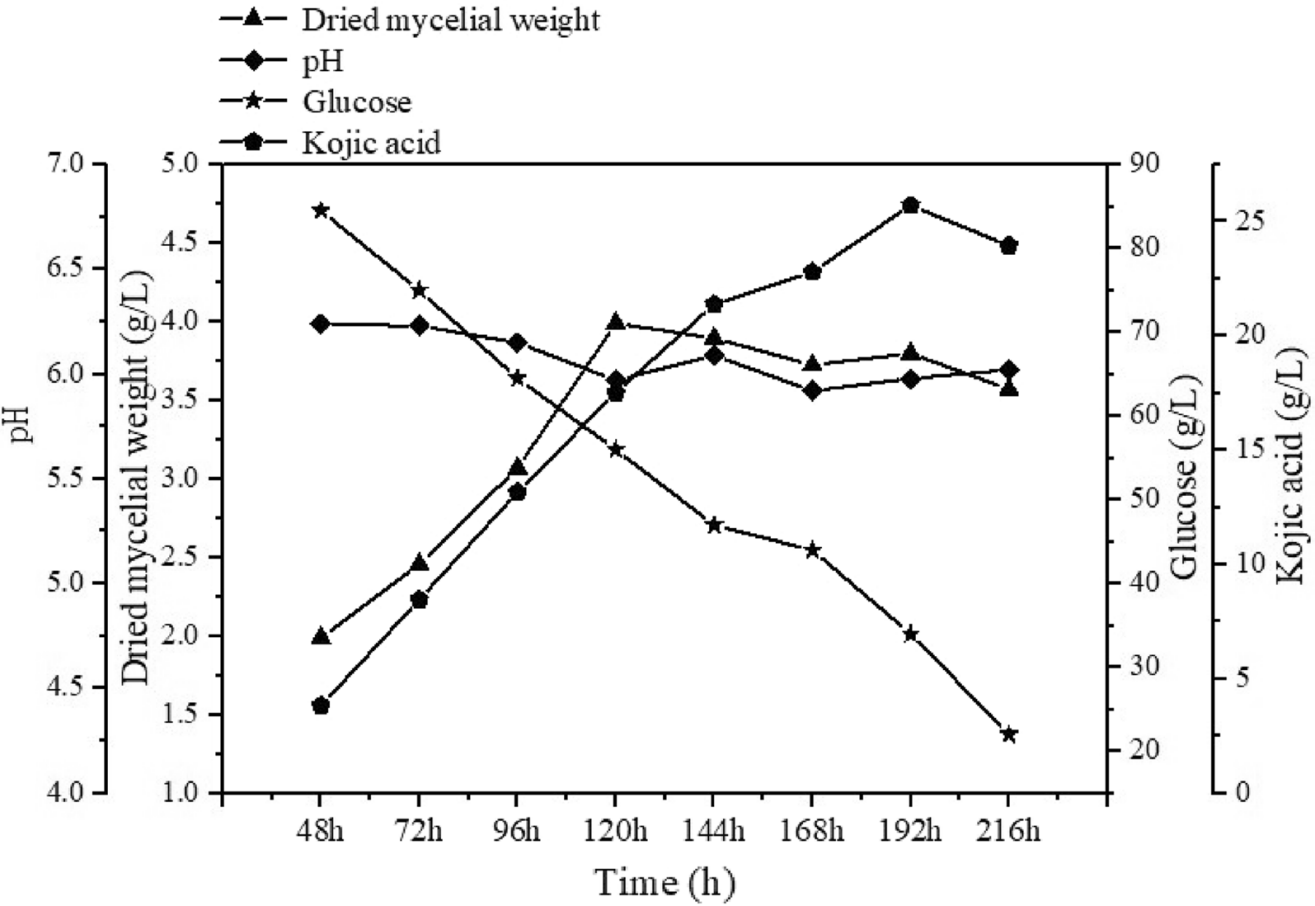
Kinetics of cell growth and kojic acid production by *A. niger* S2435 in 2 L controlled batch bioreactors. KA production, dry cell weight and residual glucose were determined. The results shown are from a single representative experiment

In this study, we also evaluated KA production in a pH controlled bioreactor using the *A. niger* strain S2435 (OE*kojA*, Δ*nrkC*), which contains the least genetic modification and displays efficient KA production activity in shake flask cultivations. The bioreactor was operated at pH 6.0 by adding HCl or NaOH as needed based on pH sensor feedback. The same medium used in shake flask cultivations, but without the addition of MES, was used in the bioreactor. As shown in Fig. 6, cell growth reached its maximum after 5 days and obvious KA accumulation can be detected at48 hours after inoculation, increasing steadily to reach 21.39 g/L after 6 days of fermentation. After that, KA productivity decreased and the titer increased slowly, reaching a maximum of 25.71 g/L after 8 days of fermentation. A similar trend was observed in glucose uptake, with the rate increasing at 48 h after inoculation and remaining constant until the 6th day of fermentation. After that, the glucose consuming rate decreased and 34 g/L of glucose still remained after 8 days of fermentation when the KA titer reached its maximum. After 7 days of cultivation, the bioreactor fermentation with pH control (22.80 g/L) had a higher titer than MES-buffered shaking flask fermentation (20.29 g/L), indicating that MES supplementation can be avoided in controlled bioreactor fermentation. However, after 8 days of fermentation in controlled aerobic batch culture, only 66% of glucose was consumed, and only 49.4% of the consumed glucose was converted to KA, indicating the need for further optimization of fermentation conditions such as medium composition, pH, and dissolved oxygen to improve KA yield in *A. niger*.

## Discussion

To date, production of KA in a heterologous host has not been reported, mainly due to the lack of clarity surrounding the biosynthesis pathway of KA. More than ten years have passed since three genes of *kojA*, *kojR* and *kojT* were identified to be involved in the KA biosynthesis process in *A. oryzae* [13]. However, to this day, no biosynthetic intermediates have been identified in the KA biosynthesis process, and the exact number of genes essential for KA production remains unknown. Based on the structural differences between glucose and KA, it is believed that at least one oxidation step (CHOH → CO) and two dehydration steps are required for the conversion of glucose to KA (though the exact order is unknown). Therefore, it has been predicted that at most two or three enzymes are needed for KA biosynthesis [1]. KA production is limited to a small number of species within *Aspergillus*, *Acetobacter,* and *Penicillium* [1]. *A. niger* does not produce any detectable KA. In this study, we report for the first time the heterologous production of KA in *A. niger* by introducing the *kojA* gene from *A. oryzae*. The protein encoded by *kojA* is predicted to be a FAD-dependent oxidoreductase. It is unlikely that KojA has the activity for the full transformation process from glucose to KA. Our study results suggest the availability of the direct precursor for the reaction catalyzed by KojA in *A. niger*. Further studies on the KojA-involved reaction in *A. niger* will contribute to a better understanding of the KA biosynthesis pathway.

Our finding that the introduction of *kojA* in *A. niger* results in KA production suggests the presence of an endogenous transporter for exporting KA in the organism. AO090113000138 (*kojT*), a gene encoding a MFS transporter, was reported to be the major transporter gene responsible for KA transportation in *A. oryzae* [13]. Upon deletion of *ASPNIDRAFT_43217*, the closest homolog of *kojT* in *A. niger*, there were no significant changes in the KA yield of the resulting gene deletion strain compared to that of the parent strain S2132. This suggests that other genes in *A. niger* play a more important role in transporting KA out of the cell. A blastP analysis showed that 6 more homologs with 60% or higher protein sequence similarities KojT to exist in *A. niger*'s genome (ASPNIDRAFT_132090, ASPNIDRAFT_174815, ASPNIDRAFT_181773, ASPNIDRAFT_183073, ASPNIDRAFT_207820, ASPNIDRAFT_39368). Further genetic studies on these candidate genes will be helpful in identifying all KA transporter encoding genes in *A. niger*.

Of the 13 genes in the putative KA biosynthetic gene cluster (from AO090113000132 to AO090113000145 in *A. oryzae* genome), besides the three closely linked genes *kojA*, *kojR*, and *kojT*, *AoKap4* (AO090113000139) and *Aokap6* (AO090113000133) were reported to also contribute to KA production in *A. oryzae* [19, 20]. *AoKap4* and *Aokap6*, encoding an MFS protein and a protein with unknown function, respectively, were reported to positively regulate KA production upstream of *kojT* and *kojA* in *A. oryzae* [19, 20]*.* However, deletion of *ASPNIDRAFT_53284* (the closest homolog of *AoKap4)* and *ASPNIDRAFT_171597* (the closest homolog of *Aokap6*) in *A. niger* S2132 resulted in similar KA production as the parent strain S2132. These findings suggest that regulation patterns for KA production vary between the native producer *A. oryzae* and the engineered KA producer *A. niger* S2132.

In this study, we identified four genes (*nrkA*, *nrkB*, *nrkC* and *nrkD*) that negatively regulate KA production in *A. niger* after screening a library composed of 10 different mutant strains. Our study showed that single knockout (or knockdown) of the four negative regulators leads to increased KA production in the resulting strain, while the combined knockout (knockdown) of all four genes does not further enhance KA production, suggesting that the four genes may participate in a shared biological process that affects the precursor supply or pathway gene expression for KA production *in A. niger*. Among the four negative regulator encoding genes, *nrkB* encodes a putative protein containing a GAL4-like Zn2Cys6 binuclear cluster DNA-binding domain and a fungal_TF_MHR domain. A similar domain composition is present in a large family of fungal zinc cluster transcription factors [33]. Considering that *kojA* in these KA-producing *A. niger* strains is driven by P*gpdA*, a constitutive promoter widely used in *A. niger* [28], we speculated that NrkB might regulate other unknown gene(s) which is involved in the biosynthesis of KA. *nrkD* encodes a sulfatase domain-containing protein. Sulfatases are enzymes that can catalyze the hydrolysis of sulfate ester bonds of a wide variety of substrates [34]. The remaining two genes (*nrkA* and *nrkC*) encode proteins with unknown functions that do not show any similarity to characterized proteins. The variable functions of the four genes indicated the complex regulation mechanism of KA biosynthesis in *A. niger*. More studies are needed to clarify the exact regulation mechanism behind this. Further research to elucidate the regulation mechanisms and functions of the four negative regulators is ongoing in our laboratory.

KA has various applications in fields such as the cosmetic industry, medicine, and food industry [3]. To meet the increasing market demand, it is crucial to optimize KA production by seeking alternatives that are more economical and have a higher production yield than current *A. oryzae*-based methods. To the best of our knowledge, our work in this study represents the first demonstration of KA production in a heterologous host. *A. niger* is one of the most important industrial filamentous fungal species. It is able to grow in a wide temperature range of 6 °C–47 °C and over an extremely wide pH range of 1.4–9.0, and it is able to ferment various cheap raw materials [25]. *A. niger* has shown advantages over other microorganisms for the commercial production of organic acids including citric acid and gluconic acid [23, 35]. In this study, we show that highly efficient KA-producing *A. niger* strains can be obtained through just two steps of genetic manipulation: the introduction of a foreign gene (*kojA*) plus the knockout (or knockdown) of an endogenous gene (*nrkA*, *nrkB*, *nrkC* or *nrkD*). As shown in Fig. 6, the KA titer of the engineered *A. niger* can reach up to 25.71 g/L, which is superior to most wild-type KA-producing strains [36]. It should be noted that the acid production medium used in this study was modified from the medium used in *A. oryzae* [13], and may not be optimal for *A. niger.* As shown in Fig. 6, glucose was not fully consumed during batch fermentation. Further research into the fermentation process optimization is ongoing. The results of our study strongly support the notion that *A. niger*-based cell factories have the potential to create industrial strains for cost-effective KA production.

## Conclusion

In this study, we demonstrate the successful reconstitution of the KA biosynthesis pathway in the heterologous host of *A. niger* by introducing the *kojA* gene from *A. oryzae*. Using the KA-producing *A. niger* strain (OE*kojA*) as a platform, we constructed a mutant library consisting of 10 mutant strains, including 8 gene deletion strains and 2 RNAi strains. Through screening of this mutant library, we identified four genes (*nrkA*, *nrkB*, *nrkC*, and *nrkD*) that function in the negative regulation of KA production. The best-performing strain (OE*kojA*, Δ*nrkC*) achieved a KA titer of 20.22 g/L after 7 days of fermentation in a shaking flask. This efficient KA production was also maintained when the strain was cultivated in MES-free medium in a controlled batch bioreactor, reaching a titer of 25.71 g/L after 8 days of fermentation. These results demonstrate that the engineered KA-producing *A. niger* can serve as a useful platform for the study of KA biosynthesis and regulation, and that *A. niger*-based cell factories have significant potential for the cost-effective production of KA.

## Methods

### Strains and growth conditions.

All strains used in this study are listed in in Table 2. The *A. niger* strain S834, derived from *A. niger* ATCC 1015, was used as the parent strain [29]. All other transformants in the study were derived from *A. niger* S834. *A. niger* strains were cultured at 28 °C on potato dextrose agar medium (PDA) supplemented with 250 μg/mL hygromycin B when required [38]. Complete medium (CM) was used for transformant screening, and minimal medium (MM) was used for selecting the glufosinate resistance marker (bar) and inducing the elimination of the hygromycin B phosphotransferase gene (*hph*) cassette (*lox*P-*hph*-*lox*P) integrated into the genomes of transformants [37]. *Escherichia coli* JM109, used for constructing and amplifying plasmids, was grown at 37 °C in Luria Bertani media (LB) supplemented with 100 μg/mL kanamycin as needed. *Agrobacterium tumefaciens* AGL-1, used for *Agrobacterium*-mediated transformation (AMT) of *A. niger*, was grown at 28 °C on LB supplemented with 100 μg/mL kanamycin [37]. The KA fermentation medium used in shake flask cultivation (consisting of 10% glucose, 0.25% yeast extract, 0.1% K_2_HPO_4_, 0.05% MgSO_4_–7H_2_O, 0.75 M 2-morpholinoethanesulphonic acid (MES), pH 6.0) was modified from the medium used for KA production in *A. oryzae* [13].

**Table 2:** Strains and plasmids used in this study

Strain or plasmid	Genotype/phenotype^a^	Source
Strains		
S834	Tet-On::*cre*, ∆*oahA*, ∆*cexA*, hyg^S^	[29]
S1991	Tet-On::*cre*, ∆*oahA*, ∆*cexA*, OE*kojA*, hyg^R^	This study
S2132	Tet-On::*cre*, ∆*oahA*, ∆*cexA*, OE*kojA*, hyg^S^	This study
S2624	Tet-On::*cre*, ∆*oahA*, ∆*cexA*, OE*kojA*, ∆*ASPNIDRAFT_50239*, hyg^R^	This study
S2922	Tet-On::*cre*, ∆*oahA*, ∆*cexA*, OE*kojA*, ∆*ASPNIDRAFT_171597*, hyg^R^	This study
S2930	Tet-On::*cre*, ∆*oahA*, ∆*cexA*, OE*kojA*, RNAi-*ASPNIDRAFT_42619*, hyg^R^	This study
S2924	Tet-On::*cre*, ∆*oahA*, ∆*cexA*, OE*kojA*, ∆*ASPNIDRAFT_189096*, hyg^R^	This study
S2929	Tet-On::*cre*, ∆*oahA*, ∆*cexA*, OE*kojA*, ∆*ASPNIDRAFT_43217*, hyg^R^	This study
S2626	Tet-On::*cre*, ∆*oahA*, ∆*cexA*, OE*kojA*, ∆*ASPNIDRAFT_53284*, hyg^R^	This study
S2933	Tet-On::*cre*, ∆*oahA*, ∆*cexA*, OE*kojA*, RNAi-*ASPNIDRAFT_56871*, hyg^R^	This study
S2430	Tet-On::*cre*, ∆*oahA*, ∆*cexA*, OE*kojA*, ∆*ASPNIDRAFT_209619*, hyg^R^	This study
S2435	Tet-On::*cre*, ∆*oahA*, ∆*cexA*, OE*kojA*, ∆*ASPNIDRAFT_186610*, hyg^R^	This study
S2437	Tet-On::*cre*, ∆*oahA*, ∆*cexA*, OE*kojA*, ∆*ASPNIDRAFT_131173*, hyg^*R*^	This study
S2743	Tet-On::*cre*, ∆*oahA*, ∆*cexA*, OE*kojA*, ∆*ASPNIDRAFT_186610*, hyg^S^	This study
S2684	Tet-On::*cre*, ∆*oahA*, ∆*cexA*, OE*kojA*, ∆*ASPNIDRAFT_186610*, ∆*ASPNIDRAFT*_131173, hyg^R^	This study
S2991	Tet-On::*cre*, ∆*oahA*, ∆*cexA*, OE*kojA*, ∆*ASPNIDRAFT_186610*, ∆*ASPNIDRAFT_131173*, hyg^S^	This study
S3058	Tet-On::*cre*, ∆*oahA*, ∆*cexA*, OE*kojA*, ∆*ASPNIDRAFT_186610*, ∆*ASPNIDRAFT_131173*, RNAi-*ASPNIDRAFT_56871,* hyg^R^	This study
S3067	Tet-On::*cre*, ∆*oahA*, ∆*cexA*, OE*kojA*, ∆*ASPNIDRAFT_186610*, ∆*ASPNIDRAFT_131173*, RNAi-*ASPNIDRAFT_56871*, hyg^S^	This study
S3119	Tet-On::*cre*, ∆*oahA*, ∆*cexA*, OE*kojA*, ∆*ASPNIDRAFT_186610,* ∆*ASPNIDRAFT_131173*, RNAi-*ASPNIDRAFT_56871*, *RNAi-ASPNIDRAFT_209619*, hyg^R^	This study
Plasmids		
pLH454	*lox*P-*hph*-*lox*P, P*gpdA*, T*trpC*, hyg^R^, kan^R^	[31]
pLH509	*lox*P-*hph*-*lox*P, P*pkiA*, T*trpC*, hyg^R^, kan^R^	[24]
pLH594	*lox*P-*hph*-*lox*P, P*gpdA*, T*trpC*, hyg^R^, ppt^R^, kan^R^	[37]
pLH1081	P*gpdA*::*kojA*, *lox*P-*hph*-*lox*P*,* hyg^R^, kan^R^	This study
pLH1453	*lox*P-*hph*-*lox*P, P*pkiA::gfp* spacer, T*trpC*, hyg^R^, kan^R^	this study
pLH1527	*ASPNIDRAFT_50239*::*lox*P-*hph*-*lox*P, hyg^R^, ppt^R^, kan^R^	This study
pLH1735	*ASPNIDRAFT_171597*::*lox*P-*hph*-*lox*P, hyg^R^, ppt^R^, kan^R^	This study
pLH1738	RNAi cassette targeting *ASPNIDRAFT_42619*, *loxP*-*hph*-*loxP*, hyg^R^, kan^R^	This study
pLH1736	*ASPNIDRAFT_189096*::*lox*P-*hph*-*lox*P, hyg^R^, ppt^R^, kan^R^	This study
pLH1737	*ASPNIDRAFT_43217*::*lox*P-*hph*-*lox*P, hyg^R^, ppt^R^, kan^R^	This study
pLH1526	*ASPNIDRAFT_53284*::*lox*P-*hph*-*lox*P, hyg^R^, ppt^R^, kan^R^	This study
pLH1739	RNAi cassette targeting *ASPNIDRAFT_56871*, *lox*P-*hph*-*lox*P, hyg^R^, kan^R^	This study
pLH1496	*ASPNIDRAFT_209619*::*lox*P-*hph*-*lox*P, hyg^R^, ppt^R^, kan^R^	This study
pLH1803	RNAi cassette targeting *ASPNIDRAFT_209619, lox*P-*hph*-*lox*P, *hyg*^R^, *kan*^R^	This study
pLH1497	*ASPNIDRAFT_186610*::*lox*P-*hph*-*lox*P, hyg^R^, ppt^R^, kan^R^	This study
pLH1498	*ASPNIDRAFT_131173*::*lox*P-*hph*-*lox*P, hyg^R^, ppt^R^, kan^R^	This study

^a^hyg^*R*^, hygromycin B resistance; hyg^*S*^, hygromycin B sensitive; ppt^*R*^, phosphinothricin resistance; kan^*R*^, kanamycin resistance

### Bioinformatic analyses

To compare the putative encoding sequences of the 13 genes (ranging from *AO090113000132* to *AO090113000145*) in the genome of *A. oryzae*, BlastP searches were conducted using the genome of the *A. niger* ATCC 1015 strain of *A. niger* (ACJE00000000.1) (https://blast.ncbi.nlm.nih.gov/Blast.cgi). The alignment sequence with the highest similarity was selected as the closest homolog for each search. Multiple sequence alignment analysis between the *A. oryzae* genes and their homologs in *A. niger* was performed using the Clustal Omega program (https://www.ebi.ac.uk/Tools/msa/clustalo/).

### Construction of plasmids

All plasmids used in this study are listed in in Table 2. All primers used in this study are listed in Additional file 1: Table S1.

*kojA* overexpression plasmid: The kojA overexpression plasmid (pLH1081) was derived from plasmid pLH454 [31] by inserting the open reading frame (ORF) of *kojA* downstream of the glyceraldehyde-3-phosphate dehydrogenase promoter (P*gpdA*) in pLH454. This was achieved through the following process: First, PCR was performed using cDNA from *Aspergillus oryzae* as the template and primer pair p3650/p3651. The PCR product was then digested with *Bam*HI and *Eco*RI and ligated into the corresponding sites of pLH454 to obtain pLH1081.

plasmids used for gene disruption: the recombinant plasmid pLH1527, used for deleting gene *ASPNIDRAFT_50239*, was constructed using pLH594 as the parent vector [37]. The construction process was the same as previously described [37]. Specifically, *A. niger* ATCC 1015 genomic DNA was used as the template to amplify the upstream and downstream fragments of *ASPNIDRAFT_50239* using PCR and the primer pair P4567/P4568 and P4569/P4570, respectively. The resulting products were then digested and ligated sequentially into the flanks of the hygromycin resistance cassette (l*ox*P-*hph*-*lox*P) in pLH594, resulting in the *ASPNIDRAFT_50239*-deletion plasmid pLH1527. The same strategy was used to construct the recombinant plasmids pLH1735, pLH1736, pLH1737, pLH1526, pLH1496, pLH1497, and pLH1498, which were used for deleting *ASPNIDRAFT_171597*, *ASPNIDRAFT_189096*, *ASPNIDRAFT_43217*, *ASPNIDRAFT_53284*, *ASPNIDRAFT_209619*, *ASPNIDRAFT_186610*, and *ASPNIDRAFT_131173*, respectively.

plasmids used for RNAi-mediated gene silencing: constructs for RNA interference (RNAi) were designed using inverted repeats of 500 bp of the coding sequence of the target gene separated by a spacer segment of green fluorescent protein (GFP) sequence, as described previously [32]. To construct the gene silencing vector, the recombinant plasmid pLH1453 was first created. It contains the hygromycin resistance cassette (*lox*P-*hph*-*lox*P), the pyruvate kinase A promoter (P*pkiA*), a spacer segment of GFP sequence, and the *trpC* terminator (T*trpC*). This was achieved by inserting a spacer segment of GFP sequence downstream of the *pkiA* promoter in pLH509 [24]. The process involved PCR using eGFP gene as the template and primer pair P3937/P3938, followed by digestion with *Kpn*I and ligation into the corresponding sites of pLH509 to obtain pLH1453. The *ASPNIDRAFT_42619* gene silencing vector pLH1738 was then constructed using pLH1453 as the parent vector. A portion of the coding sequence of *ASPNIDRAFT_42619* was PCR amplified from cDNA of *A. niger* ATCC1015 using primer pair P4237/P4238, and the antisense of *ASPNIDRAFT_42619* was PCR amplified from the same cDNA using primer pair P4239/P4240. The resulting products were digested and ligated sequentially into the flanks of the spacer segment of GFP in pLH1453 to obtain the *ASPNIDRAFT_42619* gene silencing plasmid pLH1738. This same strategy was also used for the construction of pLH1739 (for *ASPNIDRAFT_56871* silencing) and pLH1803 (for *ASPNIDRAFT_209619* silencing).

### Construction of strains

*kojA* over-expressing *A. niger* strain: the *A. niger* strain S1991 with overexpression of *kojA* was obtained by transforming pLH1081 into *A. niger* S834 through *Agrobacterium*-mediated transformation (AMT). The transforming process, as previously described by Xu et al. [31], involved introducing pLH1081 into *A. niger* S834 and screening transformants on PDA with 250 μg/mL hygromycin B. PCR analysis was then used to confirm the integration of the *kojA* expression cassette, as shown in Additional file 1: Fig. S2. The verified strain was designated as *A. niger* S1991.

Marker-less *kojA* over-expressing strain: The *A. niger* strain S2132, which over-expresses *kojA* and exhibits the hygromycin B-sensitive phenotype, was obtained by eliminating the *hph* selection marker from the genome of *A. niger* S1991 using the Cre-loxP system [31]. This process involved spreading approximately 400 conidia of S1991 on a modified MM plate supplemented with 30 μg/mL DOX, incubating at 28 °C for 5–7 days, and transferring the resulting clones to PDA plates with or without 250 μg/mL hygromycin B. The hygromycin B-sensitive colonies were selected and examined for *hph* excision using PCR analysis with primer pair *hph*-F/*hph*-R (see Additional file 1: Fig. S3). The verified *hph*-excision strain was designated as *A. niger* S2132.

Gene disruption mutants: *The ASPNIDRAFT_50239* disruption mutant (S2624) was obtained through a standard one-step gene replacement process involving homologous recombination, as previously described by Cao et al. [37]. Briefly, plasmid pLH1527 was introduced into *A. niger* S2132 through AMT, and transformants were selected on CM plates containing cefotaxime sodium (100 μg/mL), hygromycin B (250 μg/mL), ampicillin (100 μg/mL), and streptomycin (100 μg/mL) at 28 °C for 5 days. The transformants were then screened on PDA plates with hygromycin B (250 μg/mL) and MM with glufosinate ammonium (1000 μg/mL). The hygromycin B-resistant, glufosinate ammonium-sensitive transformants were considered putative disruption mutants and were further verified by PCR. The confirmed *ASPNIDRAFT_50239* disruption mutant was named S2624. The same strategy was used to create the *ASPNIDRAFT_171597* disruption mutant (S2922), *ASPNIDRAFT_189096* disruption mutant (S2924), *ASPNIDRAFT_43217* disruption mutant (S2929), *ASPNIDRAFT_53284* disruption mutant (S2626), *ASPNIDRAFT_209619* disruption mutant (S2430), *ASPNIDRAFT_186610* disruption mutant (S2435), and *ASPNIDRAFT_131173* disruption mutant (S2437) (See Additional file 1: Fig. S4).

Gene silencing mutants: The gene silencing mutants were created using the RNA interference (RNAi) technology previously described by Liu et al. [32]. To construct the RNAi strain targeting *ASPNIDRAFT_42619*, *A. niger* S2132 was transformed with plasmid pLH1738 using AMT. Transformants were screened on PDA containing hygromycin B, and PCR analysis was used to verify the integration of the RNAi cassette. The verified strain was designated as S2930. The same process was used to construct the RNAi strain targeting *ASPNIDRAFT_56871* (*A. niger* S2933). The efficient downregulation of the target gene in each construct was verified by qRT-PCR.

Multiple gene disruption (silencing) mutants: In this study, we attempted to construct disruption mutants for four negative regulator encoding genes (*nrkA*, *nrkB*, *nrkC*, *nrkD*) involved in kojic acid production. To do so, we first obtained the marker-less *nrkC* mutant (*A. niger* S2743) by eliminating the *hph* selection marker from the genome of *A. niger* S2437 (Δ*nrkC*) using the Cre-*loxP* system [31]. Starting from *A. niger* S2743 (Δ*nrkC*), we attempted to sequentially disrupt the remaining three genes using the strategy described above. However, we were only successful in disrupting *nrkD*, resulting in the strain *A. niger* S2684 (Δ*nrkC*, Δ*nrkD*). We then eliminated the *hph* selection marker from the genome of *A. niger* S2684 to obtain the marker-less strain *A. niger* S2991 (Δ*nrkC,* Δn*rkD)*. Next, we introduced the RNAi plasmid pLH1739 (targeting *nrkA*) into *A. niger* S2991 to obtain *A. niger* S3058 (RNAi-*nrkA*, Δ*nrkC*, Δ*nrkD*). We then eliminated the *hph* selection marker from the genome of *A. niger* S3058 to obtain the marker-less strain *A. niger* S3067 (RNAi-*nrkA*, Δ*nrkC*, Δ*nrkD*). Finally, we introduced the RNAi plasmid pLH1803 (targeting *nrkB*) into *A. niger* S3067 to obtain *A. niger* S3119 (RNAi-*nrkA*, RNAi-*nrkB*, Δ*nrkC*, Δ*nrkD*). The downregulation of *nrkA* and *nrkB* in *A. niger* S3067 was confirmed by qRT-PCR (Additional file 1: Fig. S5).

### RNA purification and transcription analysis

Real-time quantitative reverse transcription PCR (qRT-PCR) was performed as previously described by Cao et al. [37]. Mycelia for RNA isolation were harvested from a kojic acid production medium in shake flask cultivation. Total RNA was extracted from the shake flask culture using the E.A.N.A.TM Fungal RNA Kit (Omega Bio-tek, Inc.) according to the manufacturer’s protocol. Complementary DNA (cDNA) was synthesized from 300 ng of total RNA using the PrimeScript RT Reagent Kit (TaKaRa Biotechnology Co., Ltd.) according to the manufacturer's protocol. For real-time RT-PCR, reactions were prepared using SYBR PremixEx TaqII kit (TaKaRa Biotechnology Co., Ltd.) and run on a StepOnePlus Real-Time PCR System (Applied Biosystems). The calculated threshold cycle (Ct) for each gene amplification was normalized to the Ct of the reference gene beta-actin, and changes in gene expression levels between the selected transformants and the parental strain were calculated using the formula 2^−ΔΔCt.^ For the heterologous group, the relative gene expression levels between mutant strain and the parent strain were analyzed using the qualifed Ct (2^−ΔCt^). The primers used in this assay were designed to amplify partial cDNA sequences of the target genes and are listed in the Additional file 1: Table S1.

### Shaking flask fermentation of *A. niger*

To evaluate kojic acid production, 1 × 10^6^ conidia/mL of the engineered *A. niger* strain were inoculated into 50 mL of kojic acid fermentation medium in 250 mL Erlenmeyer flasks and incubated at 28 °C and 200 rpm for 7 days. Fermentation broths were collected at designated time points for kojic acid analysis or RNA extraction.

### Bioreactor fermentation of *A. niger*

Seed cultures were prepared by inoculating the engineered *A. niger* and incubating for 24 h at 28 °C and 200 rpm in 250 mL Erlenmeyer flasks containing 50 mL of kojic acid fermentation medium without MES (10% glucose, 0.25% yeast extract, 0.1% K_2_HPO_4_, 0.05% MgSO_4_-7H_2_O). The seed culture was then inoculated into a 1.26 L medium in a 2 L bioreactor (Baoxing Biological Engineering Co. Ltd, China) and fermented at 28 °C for 9 days using the same kojic acid fermentation medium without MES. The pH was maintained at 6.0 by automatic addition of HCl (4 M) or NaOH (4 M). The stirring speed was set at 250 rpm and the air flow rate was set at 1vvm (volume of air per volume of medium per minute). Fermentation broth was collected at intervals of 24 h, with the supernatant used for kojic acid production determination and the mycelium filtered through a pre-weighed microfiber filter and dried at 80 °C for dry cell weight measurement.

### Analytical method

Kojic acid concentration was qualitatively determined using the colorimetric method of Bentley [30] and quantitatively determined by high-performance liquid chromatography (HPLC) as described by Ariff [39]. Glucose concentration was determined using the SBA-40E biosensor analyzer (Biology Institute of Shangdong Academy of Sciences, China).

### Statistical analysis

All data points shown in this study represent the average values from three independent experiments, with error bars representing standard deviations. Statistical analysis was performed using a two-tailed Student's t-test. Statistical significance was determined as follows: *P < 0.05, **P < 0.01, ***P < 0.001.

## Supplementary Information

**Additional file 1: Table S1.** Primers used in this study. **Fig. S1.** AO090113000141 and AO090113000142 match the same homolog in *A. niger*. Protein sequence alignment between AO090113000141 *and* ASPNIDRAFT_209619 (a), Protein sequence alignment between AO090113000142 and ASPNIDRAFT_209619 (b).** Fig. S2.** PCR verification of *kojA* expression cassette insertion in *A. niger* S1991. **Fig. S3.** Construction of marker-less *kojA* overexpression strain S2132. Illustration of the elimination *hph* marker using Cre-*loxP* system (a) and PCR verification of *hph* removal in *A. niger* S2132 (b). **Fig. S4.** Construction of *A. niger* deletion mutants used in this study. Schematic diagrams of homologous recombination along with the results of PCR verification are shown for disruption of ASPNIDRAFT_50239 (a), ASPNIDRAFT_171597 (b), ASPNIDRAFT_189096 (c), ASPNIDRAFT_43217 (d), ASPNIDRAFT_53284 (e), ASPNIDRAFT_209619 (f), ASPNIDRAFT_186610 (g), ASPNIDRAFT_131173 (h). **Fig. S5.** The down-regulation of *nrkA* and *nrkB* in *A. niger* S3119. qRT-PCR analysis of *nrkA* (a) and *nrkB* (b) for the parent strain S2743 and the final construction S3119. Results were first standardized against actin, with S2743 expression set arbitrarily to 1.

## Data Availability

The datasets used and analyzed during the current study are available from the corresponding author upon reasonable request.

## Abbreviations

KA: Kojic acid
P*gpd*A: Promoter of glyceraldehyde-3-phosphate dehydrogenase gene
PpkiA: Promoter of pyruvate kinase A gene
OE: Overexpression
Ct: The calculated threshold cycle
PDA: Potato dextrose agar
LB: Luria Bertani
MES: Morpholinoethanesulfonic acid
DOX: Doxycycline
HPLC: High-performance liquid chromatography
RNAi: RNA interference
MFS: Major facilitator superfamily
