# Supplementary material for: Effective production of kojic acid in engineered Aspergillus niger
Source: Microb Cell Fact. 2023 Feb 27;22:40. doi: 10.1186/s12934-023-02038-w (PMC9969635; doi:10.1186/s12934-023-02038-w)
Supplement: Supplementary file 1 — Additional file 1: Table S1. Primers used in this study. Fig. S1. AO090113000141 and AO090113000142 match the same homolog in A. niger. Protein sequence alignment between AO090113000141 and ASPNIDRAFT_209619 (a), Protein sequence alignment between AO090113000142 and ASPNIDRAFT_209619 (b). Fig. S2. PCR verification of kojA expression cassette insertion in A. niger S1991. Fig. S3. Construction of marker-less kojA overexpression strain S2132. Illustration of the elimination hph marker using Cre-loxP system (a) and PCR verification of hph removal in A. niger S2132 (b). Fig. S4. Construction of A. niger deletion mutants used in this study. Schematic diagrams of homologous recombination along with the results of PCR verification are shown for disruption of ASPNIDRAFT_50239 (a), ASPNIDRAFT_171597 (b), ASPNIDRAFT_189096 (c), ASPNIDRAFT_43217 (d), ASPNIDRAFT_53284 (e), ASPNIDRAFT_209619 (f), ASPNIDRAFT_186610 (g), ASPNIDRAFT_131173 (h). Fig. S5. The down-regulation of nrkA and nrkB in A. niger S3119. qRT-PCR analysis of nrkA (a) and nrkB (b) for the parent strain S2743 and the final construction S3119. Results were first standardized against actin, with S2743 expression set arbitrarily to 1. [file 12934_2023_2038_MOESM1_ESM.zip › Additional file1.pptx]

## Slide 1
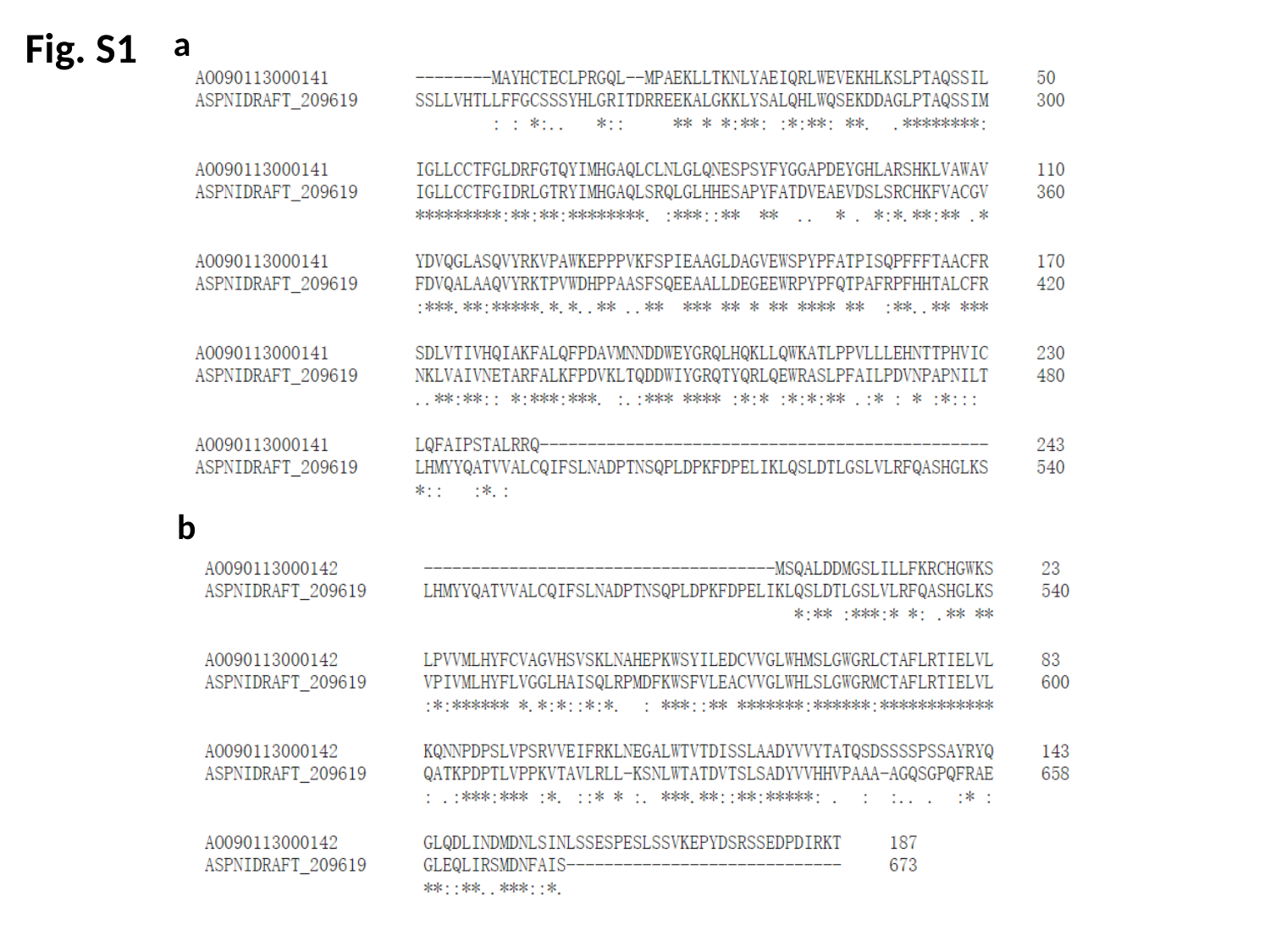

Fig. S1
a
b

## Slide 2
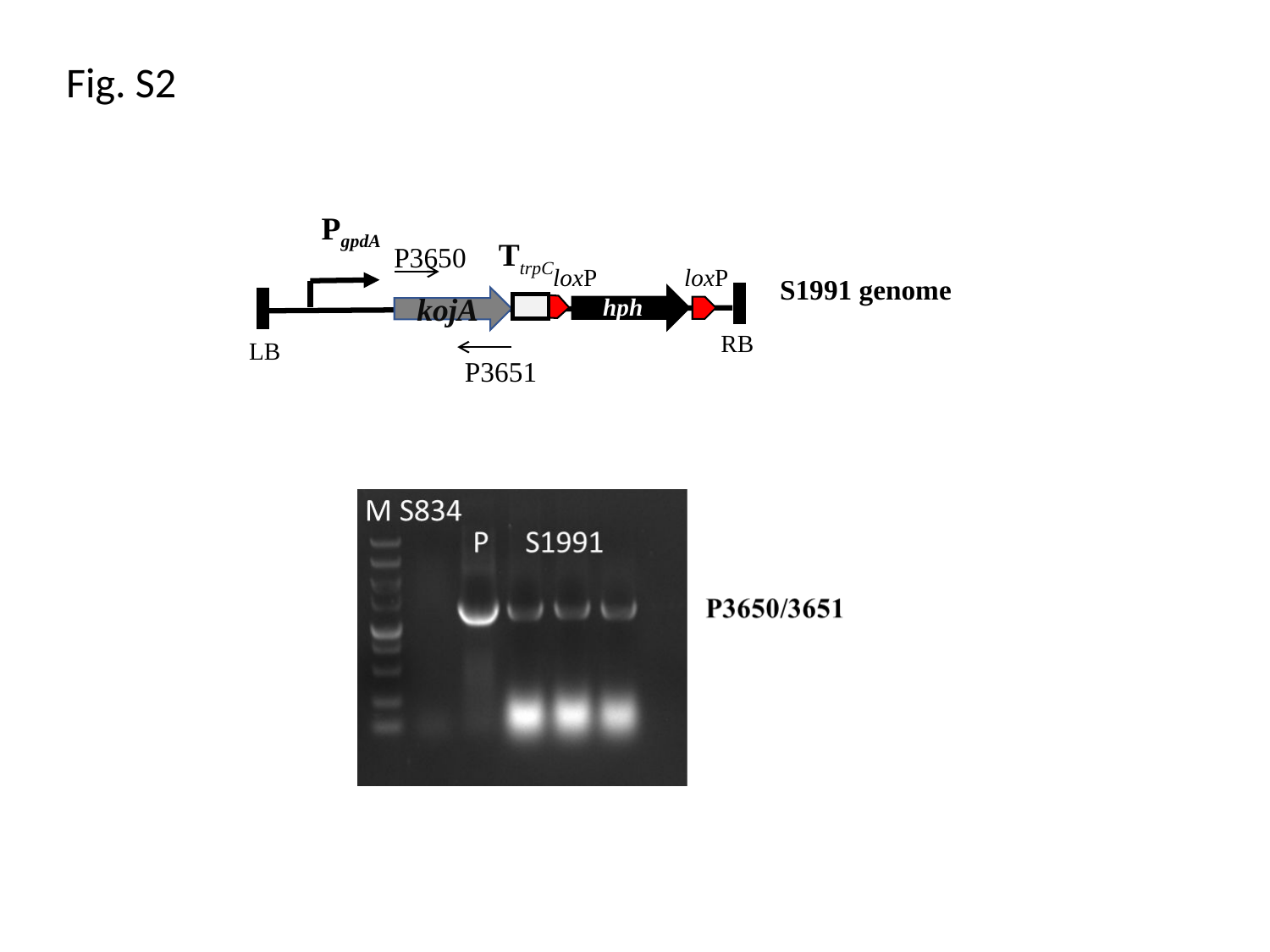

Fig. S2
PgpdA
TtrpC
P3650
loxP
loxP
S1991 genome
hph
kojA
RB
LB
P3651

## Slide 3
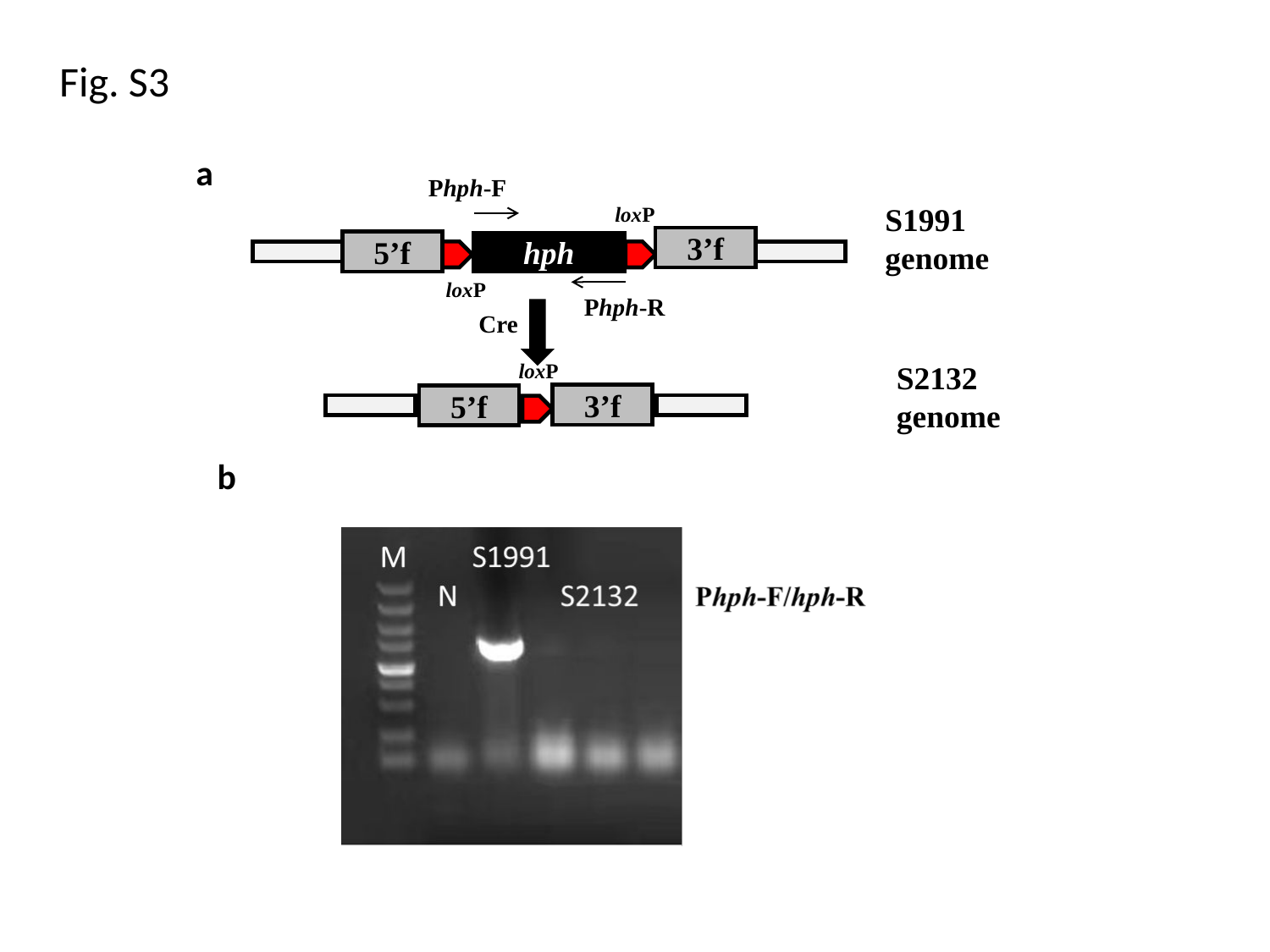

Fig. S3
a
Phph-F
S1991
genome
loxP
3’f
5’f
hph
loxP
Phph-R
Cre
S2132
genome
loxP
3’f
5’f
b

## Slide 4
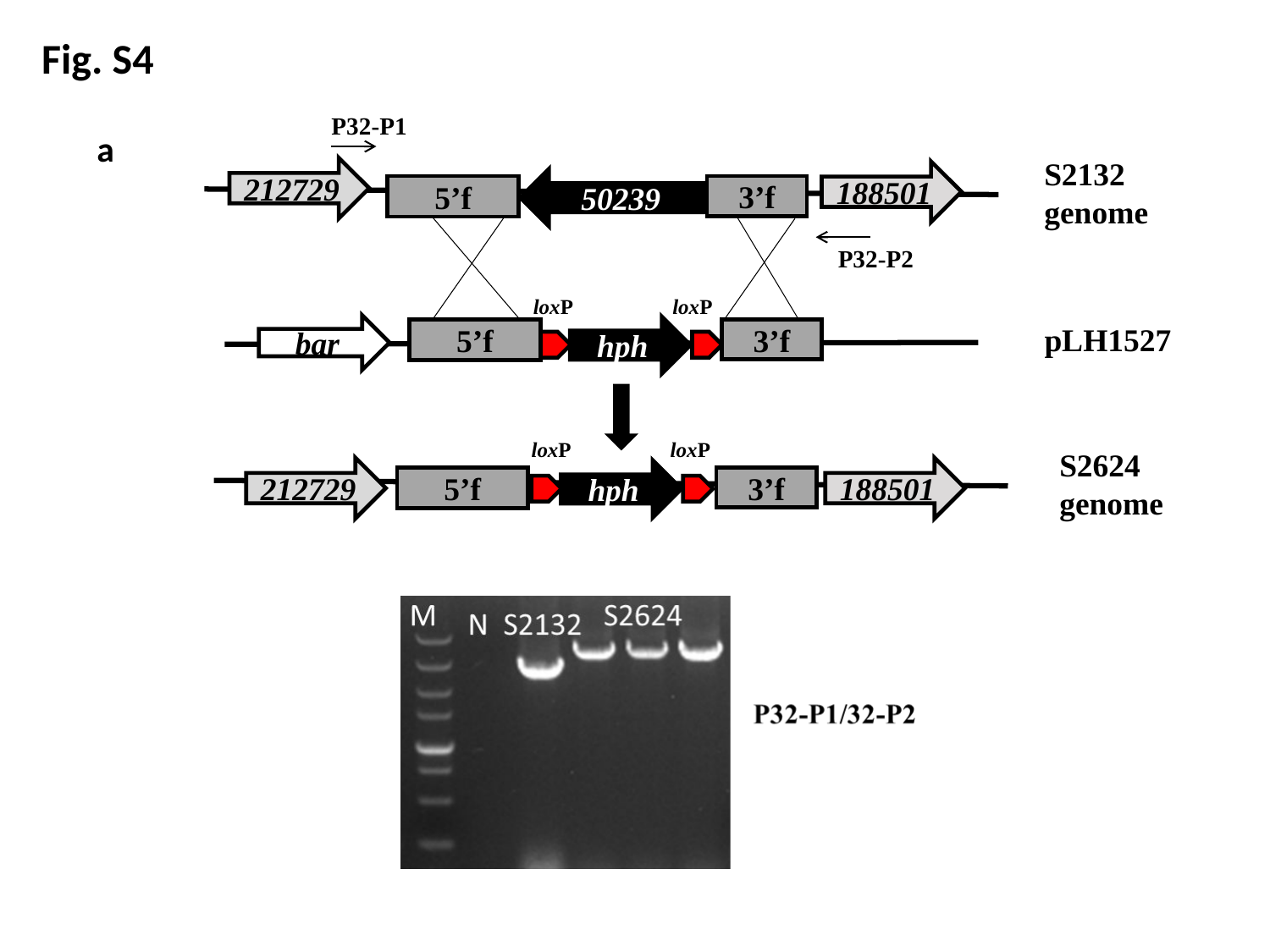

Fig. S4
P32-P1
S2132
genome
212729
188501
50239
5’f
3’f
P32-P2
loxP
loxP
pLH1527
hph
bar
3’f
5’f
loxP
loxP
S2624
genome
212729
hph
188501
3’f
5’f
a

## Slide 5
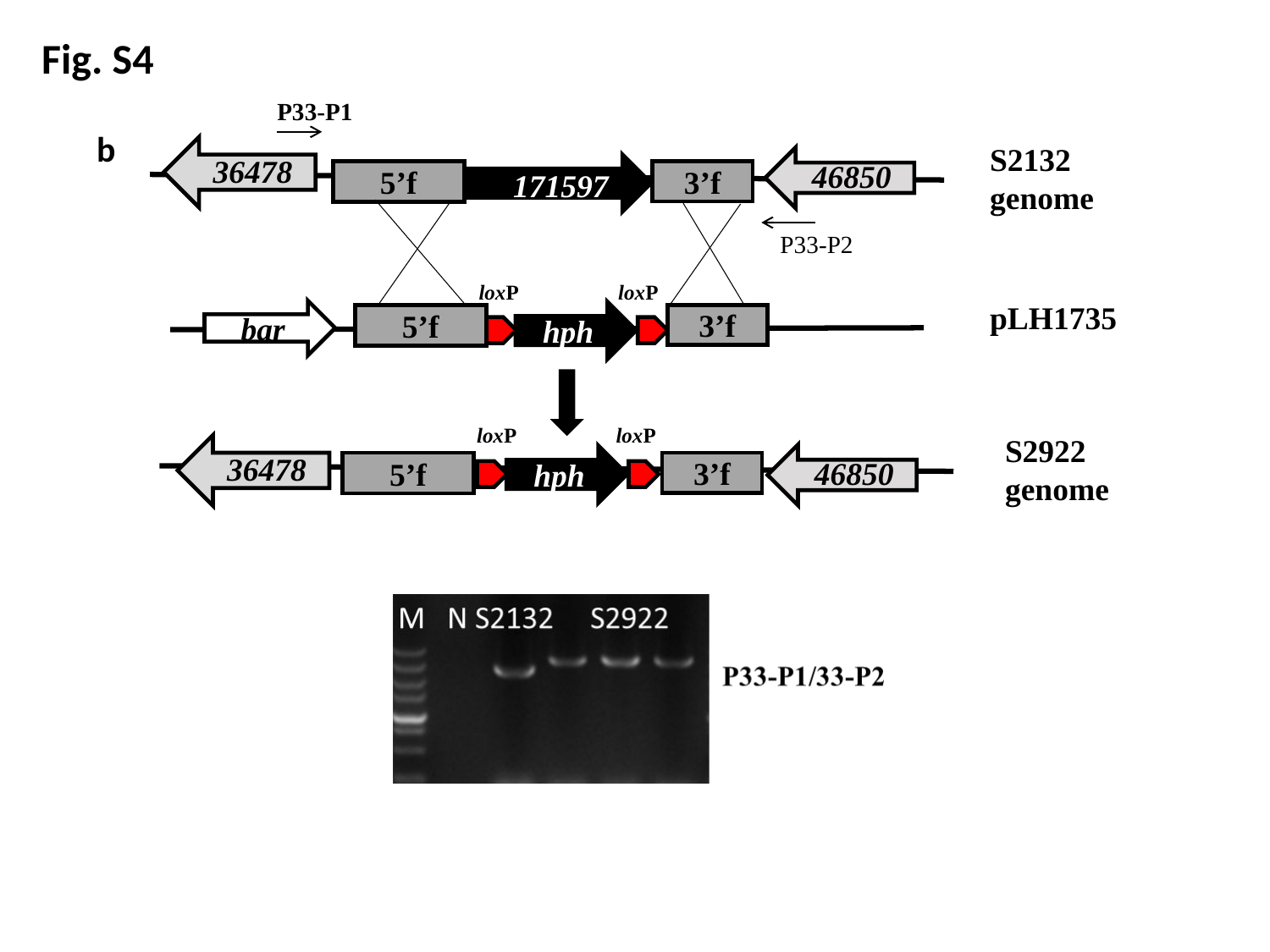

Fig. S4
P33-P1
S2132
genome
36478
46850
171597
3’f
5’f
P33-P2
loxP
loxP
pLH1735
hph
bar
3’f
5’f
loxP
loxP
S2922
genome
36478
hph
46850
3’f
5’f
b

## Slide 6
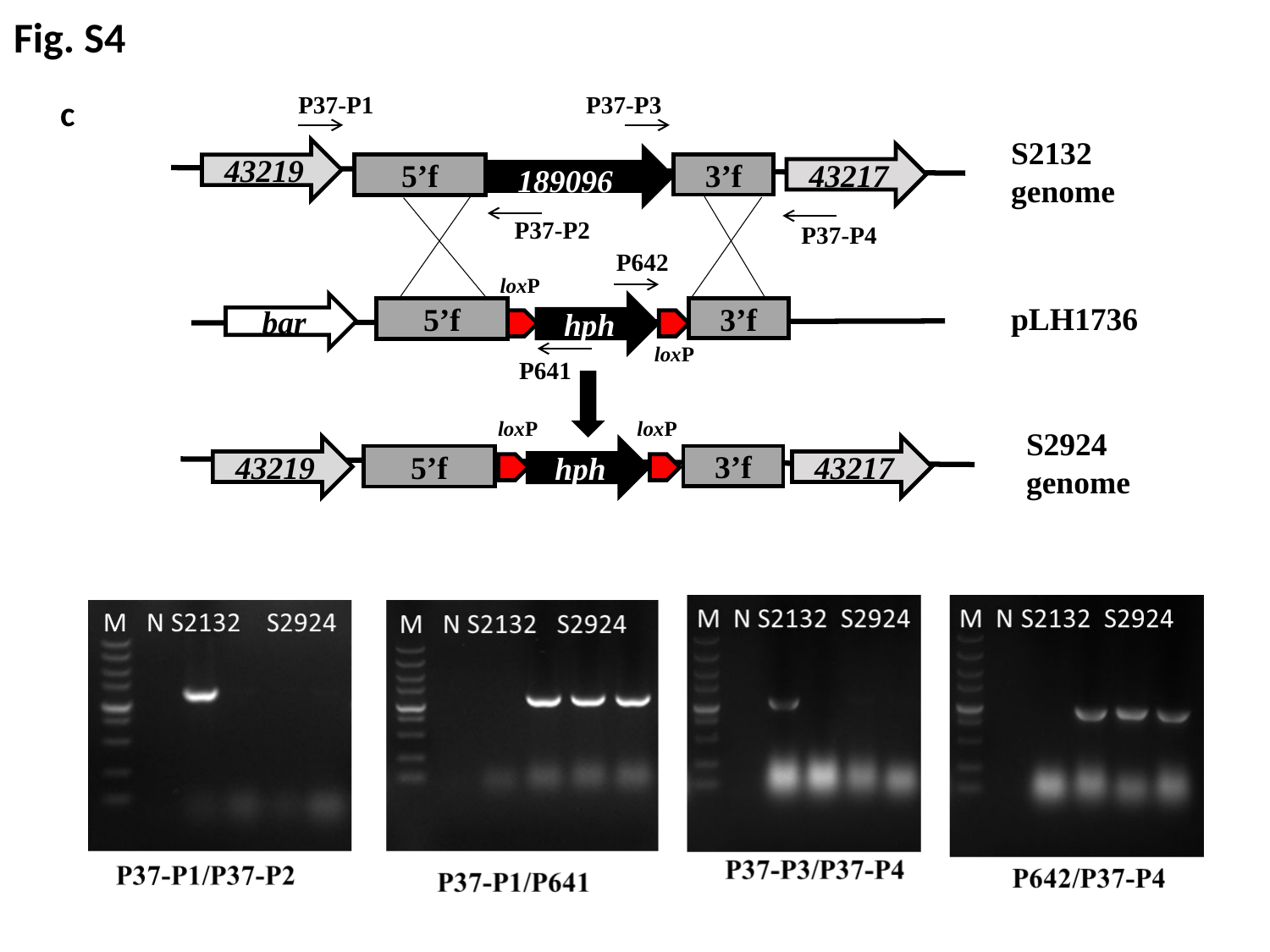

Fig. S4
P37-P1
P37-P3
S2132
genome
43219
43217
3’f
5’f
189096
P37-P2
P37-P4
P642
loxP
pLH1736
hph
bar
3’f
5’f
loxP
P641
loxP
loxP
S2924
genome
43219
hph
43217
3’f
5’f
c

## Slide 7
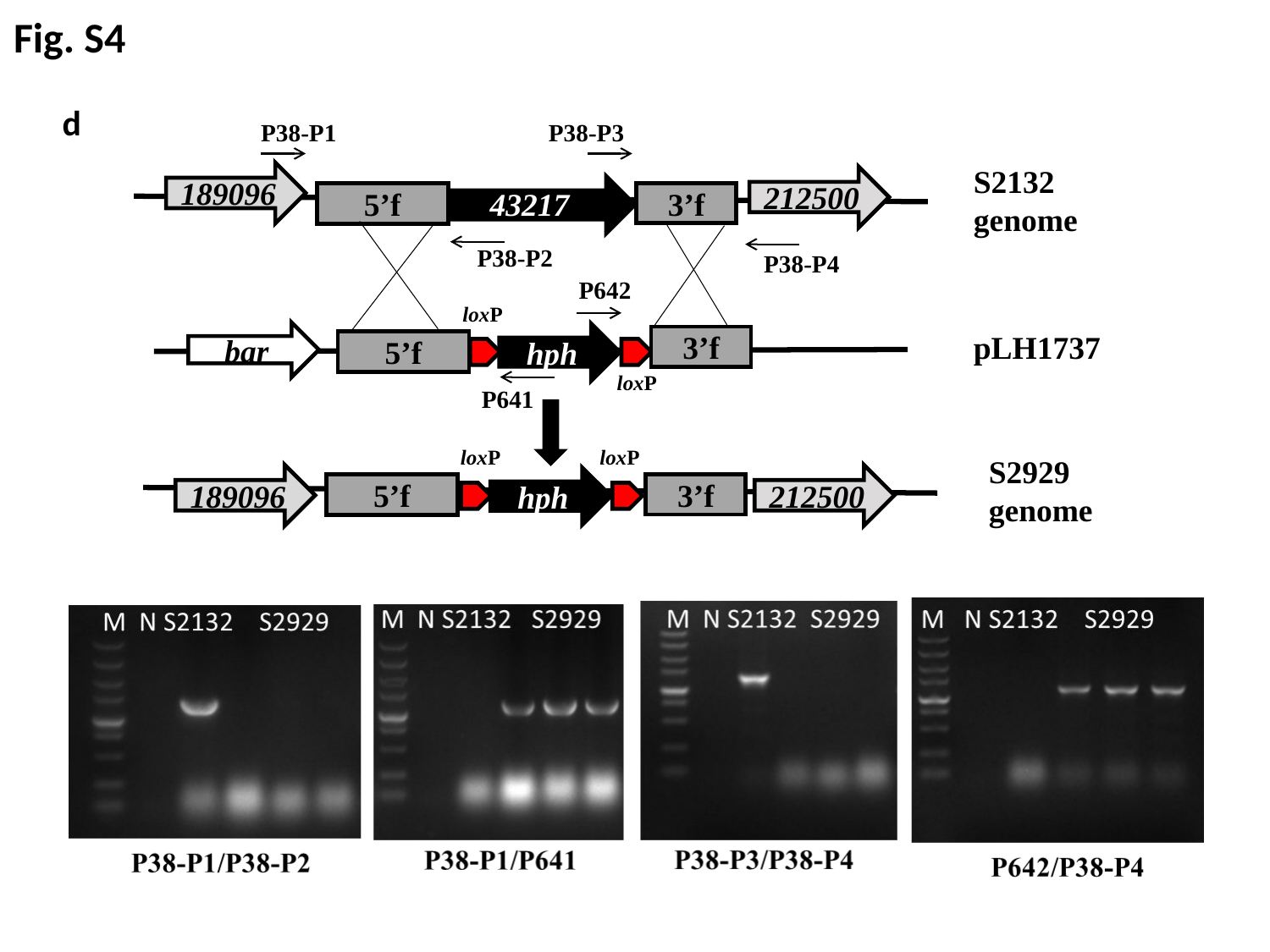

Fig. S4
d
P38-P1
P38-P3
S2132
genome
189096
212500
43217
3’f
5’f
P38-P2
P38-P4
P642
loxP
pLH1737
hph
bar
3’f
5’f
loxP
P641
loxP
loxP
S2929
genome
189096
hph
212500
3’f
5’f

## Slide 8
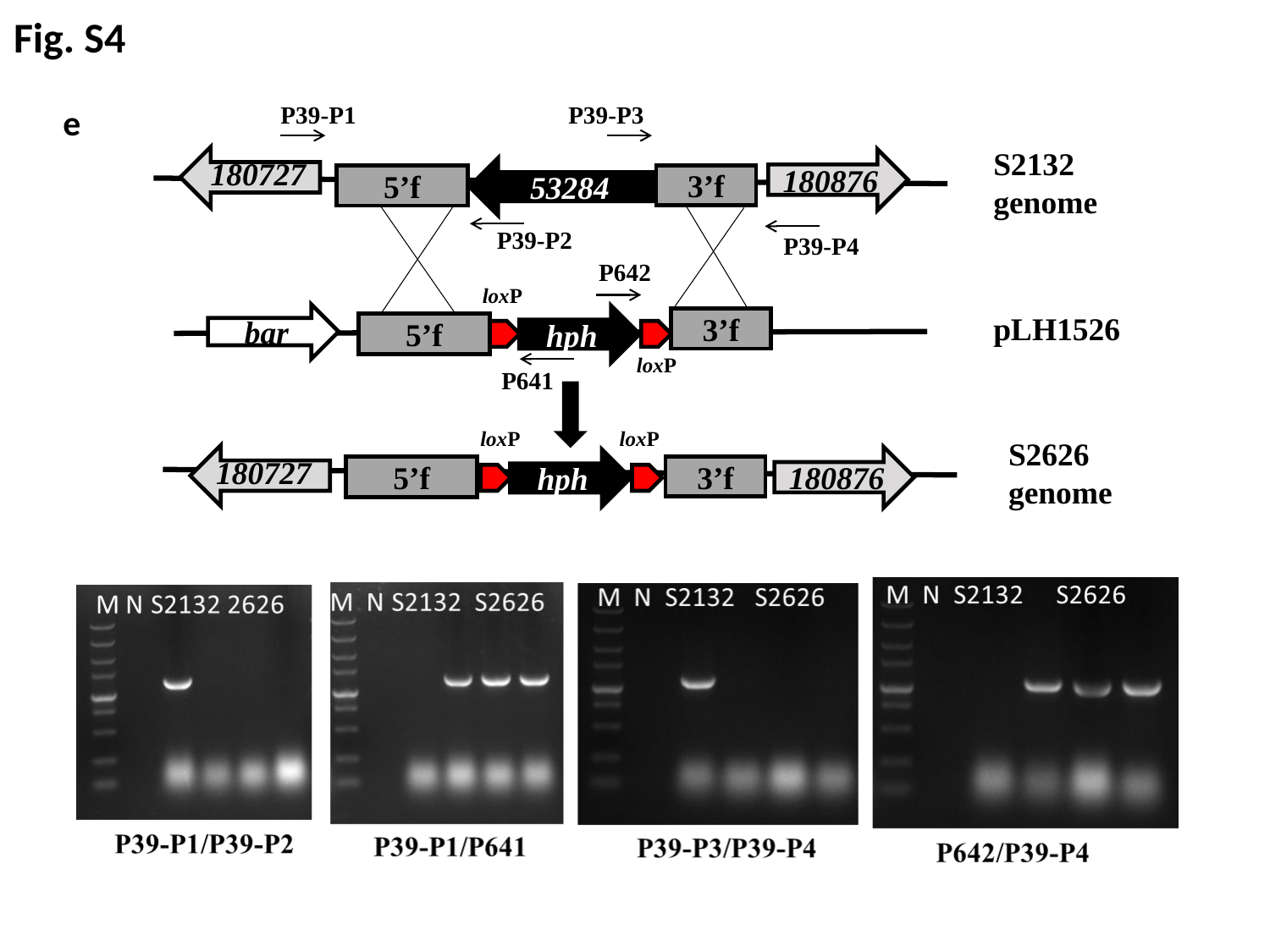

Fig. S4
P39-P1
P39-P3
S2132
genome
180727
180876
53284
3’f
5’f
P39-P2
P39-P4
P642
loxP
pLH1526
hph
bar
3’f
5’f
loxP
P641
loxP
loxP
S2626
genome
180727
hph
180876
3’f
5’f
e

## Slide 9
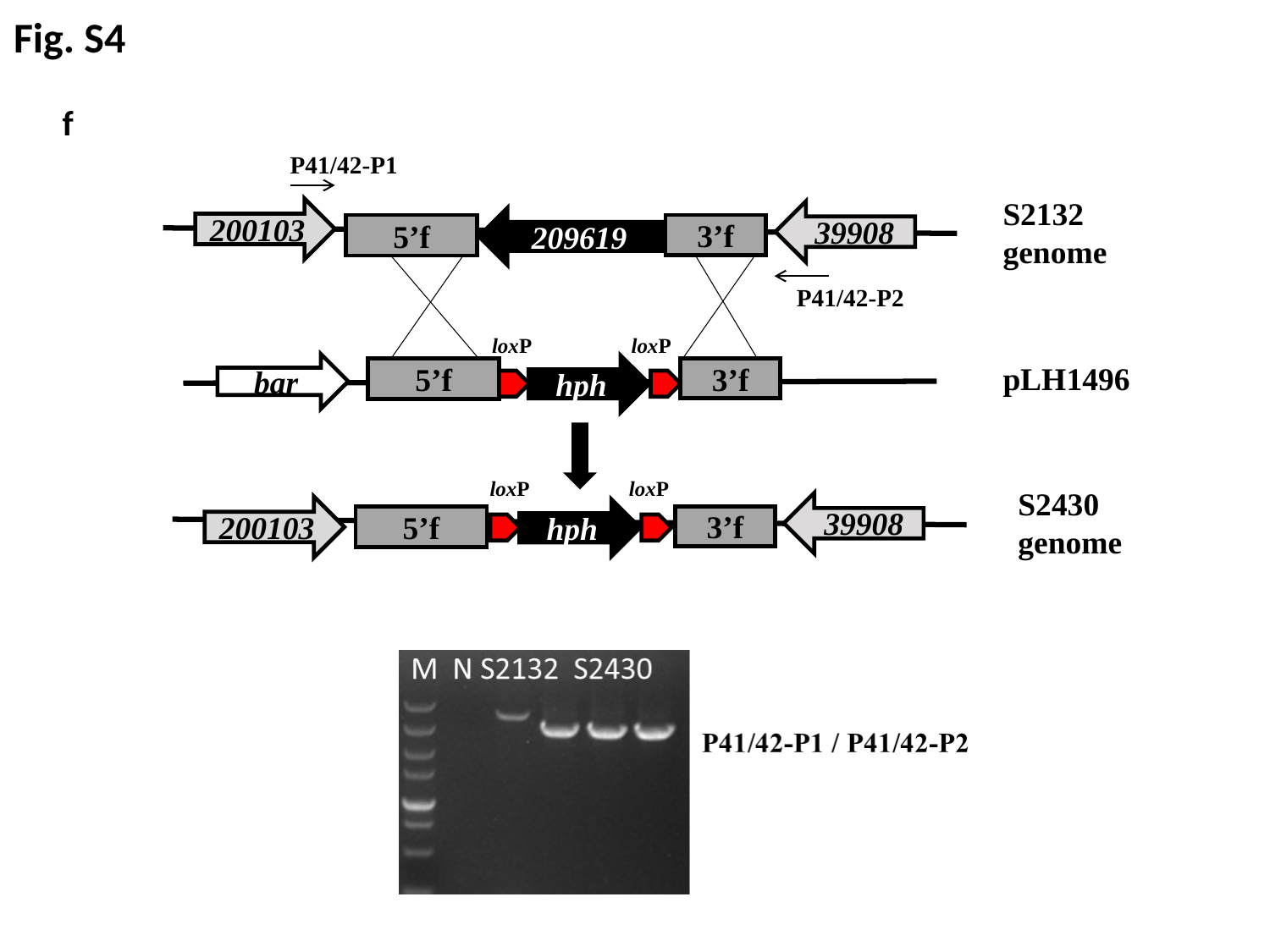

Fig. S4
f
P41/42-P1
S2132
genome
200103
209619
39908
3’f
5’f
P41/42-P2
loxP
loxP
pLH1496
hph
bar
3’f
5’f
loxP
loxP
S2430
genome
39908
200103
hph
3’f
5’f

## Slide 10
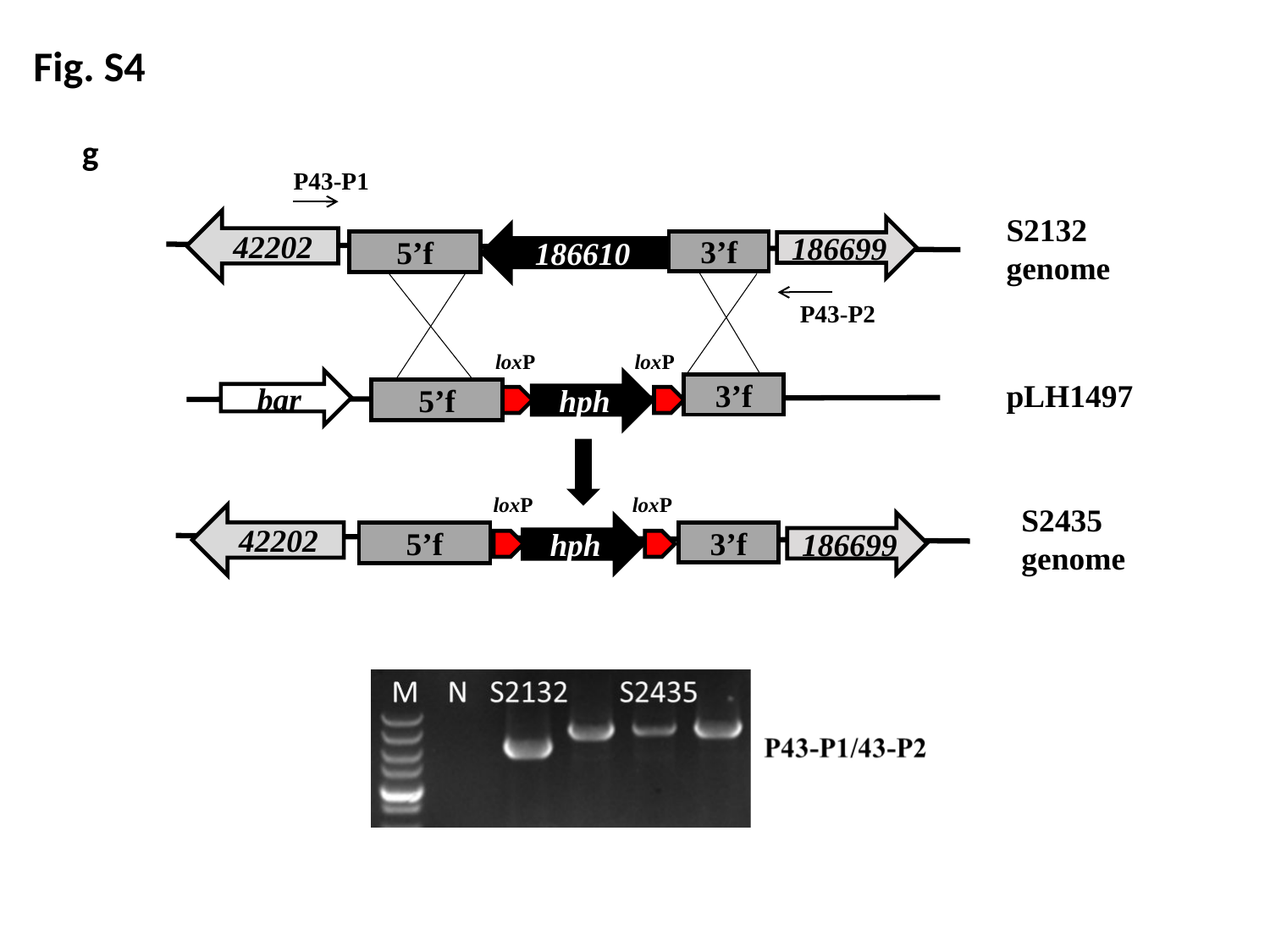

Fig. S4
g
P43-P1
S2132
genome
42202
186699
186610
3’f
5’f
P43-P2
loxP
loxP
pLH1497
hph
bar
3’f
5’f
loxP
loxP
S2435
genome
42202
hph
186699
3’f
5’f

## Slide 11
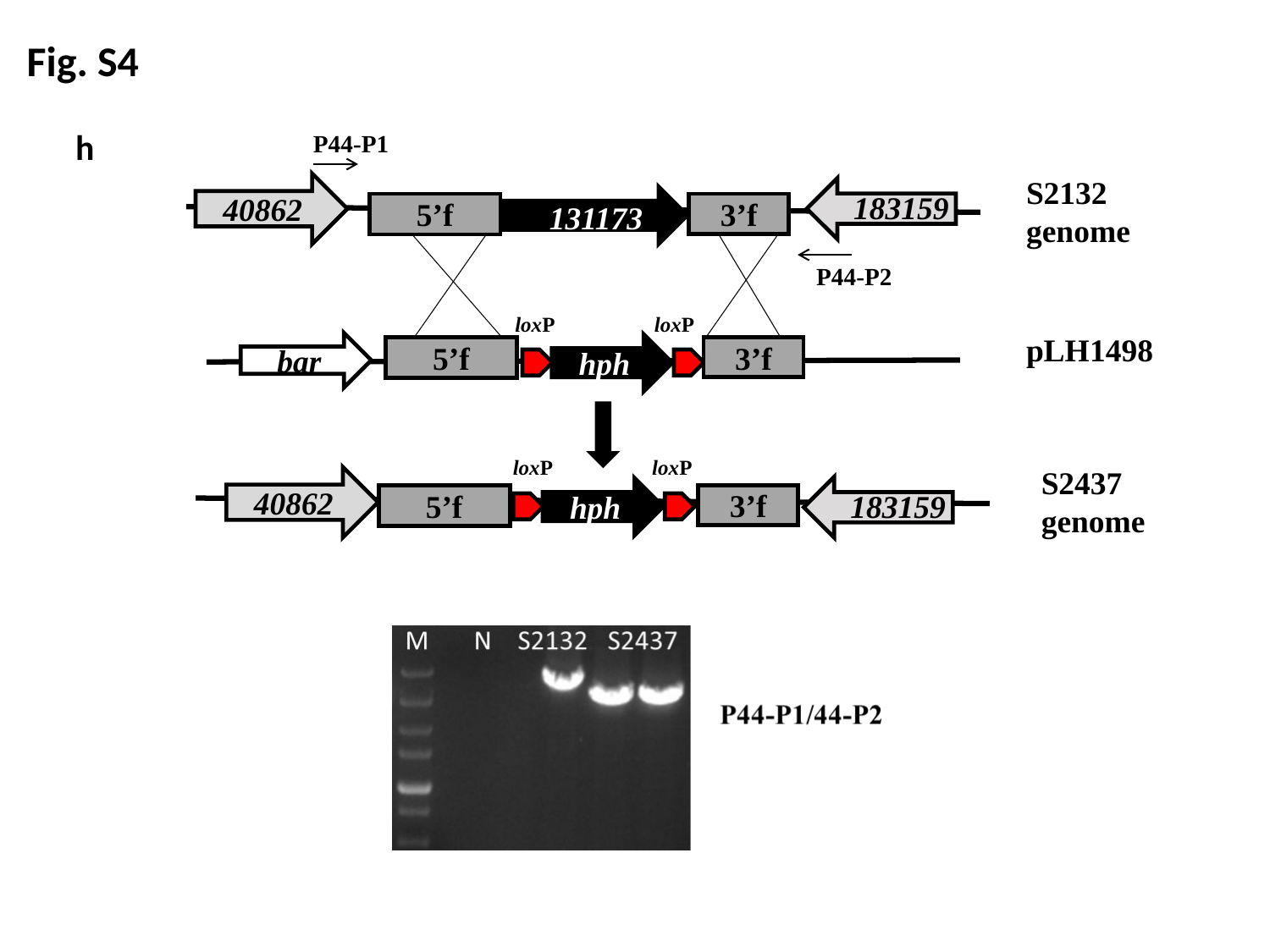

Fig. S4
h
P44-P1
S2132
genome
40862
183159
131173
3’f
5’f
P44-P2
loxP
loxP
pLH1498
hph
bar
3’f
5’f
loxP
loxP
S2437
genome
40862
hph
183159
3’f
5’f

## Slide 12
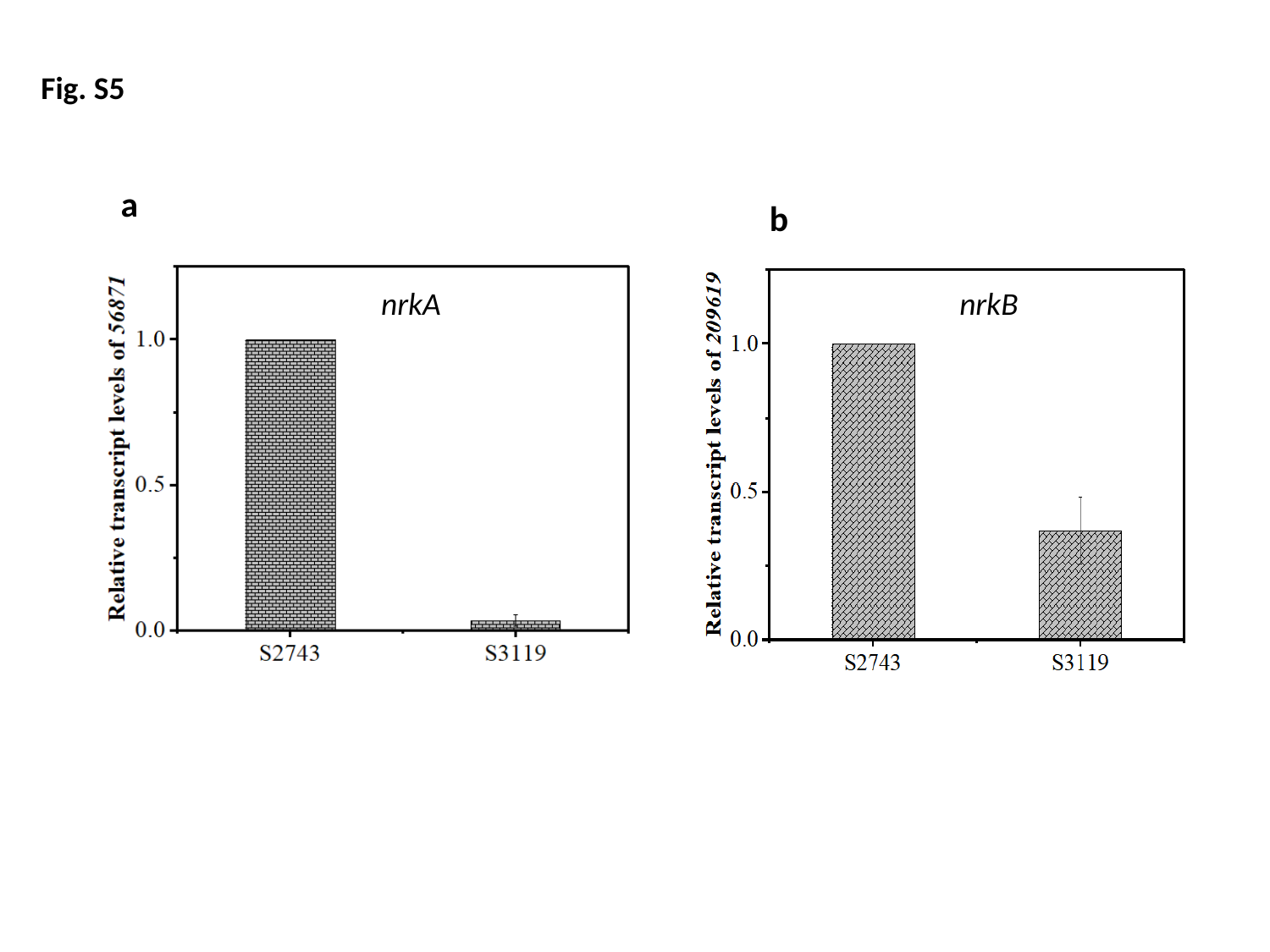

Fig. S5
a
b
nrkA
nrkB
