# Supplementary material for: Effective production of kojic acid in engineered Aspergillus niger
Source: Microb Cell Fact. 2023 Feb 27;22:40. doi: 10.1186/s12934-023-02038-w (PMC9969635; doi:10.1186/s12934-023-02038-w)
Supplement: Supplementary file 1 — Additional file 1: Table S1. Primers used in this study. Fig. S1. AO090113000141 and AO090113000142 match the same homolog in A. niger. Protein sequence alignment between AO090113000141 and ASPNIDRAFT_209619 (a), Protein sequence alignment between AO090113000142 and ASPNIDRAFT_209619 (b). Fig. S2. PCR verification of kojA expression cassette insertion in A. niger S1991. Fig. S3. Construction of marker-less kojA overexpression strain S2132. Illustration of the elimination hph marker using Cre-loxP system (a) and PCR verification of hph removal in A. niger S2132 (b). Fig. S4. Construction of A. niger deletion mutants used in this study. Schematic diagrams of homologous recombination along with the results of PCR verification are shown for disruption of ASPNIDRAFT_50239 (a), ASPNIDRAFT_171597 (b), ASPNIDRAFT_189096 (c), ASPNIDRAFT_43217 (d), ASPNIDRAFT_53284 (e), ASPNIDRAFT_209619 (f), ASPNIDRAFT_186610 (g), ASPNIDRAFT_131173 (h). Fig. S5. The down-regulation of nrkA and nrkB in A. niger S3119. qRT-PCR analysis of nrkA (a) and nrkB (b) for the parent strain S2743 and the final construction S3119. Results were first standardized against actin, with S2743 expression set arbitrarily to 1. [file 12934_2023_2038_MOESM1_ESM.zip › Table S1.docx]

Table S1 Primers used in this study

| Primer and Purpose | Sequence（5'-3'） |
| --- | --- |
| pLH1081 construction | |
| P3650 | CACATCTAAACAATGGAATTCCGTGTCGCGACACAGCTAA |
| P3651 | TCAGTAACGTTAAGTGGATCCTTAGTTTGCAGTCACTAGTGAAACAT |
| pLH1453 construction | |
| Primer3937 | ATGGAATTCGAGCTCGGTACCTCCAGGAGCGCACCATCT |
| Primer3938 | AGTGGATCCCTGCAGCTTCAGCTCGATGCGGTTC |
| pLH1527 construction | |
| P4567 | GCTCCGTAACACCCAGAATTCCATTGCTCGCAATTGGGG |
| P4568 | ATTATACGAAGTTATGGATCCGTTATCAGGGAAGGGGAGGG |
| P4569 | GCTATACGAAGTTATTCTAGAGGCAGTCGACGATCACAAG |
| P4570 | GCCAAGCTTGCATGCCTGCAGCATAGTGGGATGGTGGCAG |
| pLH1735 construction | |
| P4233 | GCTCCGTAACACCCAGAATTCCCAGTCTCGCAGCATTGTC |
| P4234 | ATTATACGAAGTTATGGATCCCATTGGAGACGGAGAATTGTGG |
| P4235 | GCTATACGAAGTTATTCTAGAGCCAGTTCGAGTCTCCCTAC |
| P4236 | TGCCTGCAGGGGCCCACTAGTCGTTGCTGCTGTTGAAAGATCG |
| pLH1738 construction | |
| P4237 | TCATCCGTCAAGATGGAATTCCGTACTTTCTAGACCGCGATGC |
| P4238 | GCTCCTGGAGGTACCGAGCTCCATCGACATCCAGAGCACCAC |
| P4239 | CGCATCGAGCTGAAGCTGCAGCATCGACATCCAGAGCACCAC |
| P4240 | ACGTTAAGTGGATCCCTGCAGCGTACTTTCTAGACCGCGATGC |
| pLH1736 construction | |
| P4241 | GCTCCGTAACACCCAGAATTCGCATGTATCCGCCTTGCTC |
| P4242 | ATTATACGAAGTTATGGATCCGAGAGAATGCGGAAGGTGC |
| P4243 | GCTATACGAAGTTATTCTAGACCATGCGTTGATCTGGCTACC |
| P4244 | CAGGGGCCCACTAGTTCTAGACGACATACTCCCACCGAATG |
| pLH1737 construction | |
| P4245 | GCTCCGTAACACCCAGAATTCGCACATCATCGAAGCAGCAG |
| P4246 | ATTATACGAAGTTATGGATCCGCATGTTGAACTGGGCAGC |
| P4247 | CGAAGTTATTCTAGAACTAGTGCCTCAATGAACTAGCACGAG |
| P4248 | GCCAAGCTTGCATGCCTGCAGGGGTGGTTATCTTGGTGCTTG |
| pLH1526 construction | |
| P4563 | GCTCCGTAACACCCAGAATTCCCGACAACAAGCAAGCTAAGC |
| P4564 | ATTATACGAAGTTATGGATCCCAAGAAGTTTGGCTTTGCAGG |
| P4565 | GCTATACGAAGTTATTCTAGAGTGCCTACCTAACTACTTGCCC |
| P4566 | GCCAAGCTTGCATGCCTGCAGCCACCAATCGAGCATTCACG |
| pLH1739 construction | |
| P4914 | TCATCCGTCAAGATGGAATTCGTCCTCCTCTTCGTCGCATC |
| P4915 | GCTCCTGGAGGTACCGAGCTCCCTTGATGCCGTTGTTGTCG |
| P4916 | CGCATCGAGCTGAAGCTGCAGCCTTGATGCCGTTGTTGTCG |
| P4917 | ACGTTAAGTGGATCCCTGCAGGTCCTCCTCTTCGTCGCATC |
| pLH1496 construction | |
| P4356 | GCTCCGTAACACCCAGAATTCCCCGTAGTTGTACCAGGACG |
| P4357 | ATTATACGAAGTTATGGATCCGCCGCAGGAAGAAAGACGA |
| P4358 | GCTATACGAAGTTATTCTAGACGGTGAGGACGGCATTCTG |
| P4359 | GCCAAGCTTGCATGCCTGCAGGAAGAGTGGTGGTTCGGTGTC |
| pLH1803 construction | |
| P4249 | TCATCCGTCAAGATGGAATTCCATGTACTATCAGGCAACGGTC |
| P4250 | GCTCCTGGAGGTACCGAGCTCGGGACATGATGCACCACATAG |
| P4251 | CGCATCGAGCTGAAGCTGCAGGGGACATGATGCACCACATAG |
| P4252 | ACGTTAAGTGGATCCCTGCAGCATGTACTATCAGGCAACGGTC |
| pLH1497 construction | |
| P4360 | GCTCCGTAACACCCAGAATTCCGGATACATCGGCCTCAAATG |
| P4361 | ATTATACGAAGTTATGGATCCGTGGCGGTTGAGTAGATAGGG |
| P4362 | GCTATACGAAGTTATTCTAGAAAGCGGAGGTGGTTAACGG |
| P4363 | CAGGGGCCCACTAGTTCTAGAGCCATCGATTACTTTGCGCAT |
| pLH1498 construction | |
| P4364 | GCTCCGTAACACCCAGAATTCCATGCTCCTTCCCTCATAACAG |
| P4365 | ATTATACGAAGTTATGGATCCGGTTTATTCACGGTGTAGCGG |
| P4366 | GCTATACGAAGTTATTCTAGAGGCTCACTCACCAACTTCACC |
| P4367 | TGCCTGCAGGGGCCCACTAGTCCTCGGACACACTCAGTACC |
| *ASPNIDRAFT_50239* deletion verification | |
| 32-P1 | GCTGCAGATCTCTCCCAAGTC |
| 32-P2 | CAGCGCTGGAGAGTACTGTC |
| *ASPNIDRAFT_171597* deletion verification | |
| 33-P1 | CGTGTCTCCTATGTCGTCG |
| 33-P2 | CTTCCTGTGCTGCATCTTGG |
| *ASPNIDRAFT_189096* deletion verification | |
| 37-P1 | CGTCGGCTAGGGATAACAG |
| 37-P2 | CAATGGGTGGTCGACTGG |
| 37-P3 | CTACGCAAGAGTACGGTTCG |
| 37-P4 | GACAACGCACAGCACACC |
| *ASPNIDRAFT_43217* deletion verification | |
| 38-P1 | GCTACTGGATGGGTCTTACG |
| 38-P2 | GCCGAGCGTCTTCATAGTC |
| 38-P3 | GTATCTCCCTACTGATGACGCC |
| 38-P4 | GCCGTGGGAATTAATGTGGG |
| *ASPNIDRAFT_53284* deletion verification | |
| 39-P1 | CATTCGACGGTAGGGAGAG |
| 39-P2 | GCAAGCGACTAAGAAGATCTGG |
| 39-P3 | GGCAACTGTGGAGGAAATAGAG |
| 39-P4 | CATGCACGGTAAGTAGAGACC |
| *ASPNIDRAFT_209619* deletion verification | |
| 41/42-P1 | CGTAGATGCATCCATTGTGTG |
| 41/42-P2 | CTGTGGGTAGTAGTGTATCGTC |
| *ASPNIDRAFT_186610* deletion verification | |
| 43-P1 | CGAGAATATCAATTTGCCTCCG |
| 43-P2 | CGCGGAAAGCATTGCTTC |
| *ASPNIDRAFT_131173* deletion verification | |
| 44-P1 | CTCCGGGTGGGAGTTGTC |
| 44-P2 | GGCTTCAGTAGAGAACCTGACC |
| Gene deletion verification (corresponding to the internal region of *hph*) | |
| P641 | CAATATCAGTTAACGTCGAC |
| P642 | GGAACCAGTTAACGTCGAAT |
| *hph* elimination verification |  |
| *hph*-F | GTGGAGGTCAACAATGAATGCC |
| *hph*-R | GTCGGTTTCCACTATCGGC |
| Verification of RNAi vectors and RNAi strains | |
| P*pkiA*-F | CTCTCTCTTCTCTCGCTCACC |
| *gfp*-P1 | CGTCGTCCTTGAAGAAGATGG |
| *gfp*-P2 | CCCTGGTGAACCGCATCG |
| T*trpC*-R | GCTGACATCGACACCAACG |
| *kojA* qRT-PCR |  |
| qPCR-*kojA*-F | CTTGACACACGGCAGTTACAC |
| qPCR-*kojA*-R | GTTACAACGTTGCTCACCTTGC |
| *ASPNIDRAFT_42619* qRT-PCR | |
| qPCR-34-F | CCAAAAGACCTACCCCGG |
| qPCR-34-R | GCGGCAGGGACTTTATCG |
| *ASPNIDRAFT_56871* qRT-PCR | |
| qPCR-40-F | GATTGTACCGGGGTGTGG |
| qPCR-40-R | GACTTTGTCGCCCGTCAG |
| *ASPNIDRAFT_209619* qRT-PCR | |
| qPCR-41/42-F | CATAACCGATACGCAGCAGAC |
| qPCR-41/42-R | CGCGGTGAGAGTTGAGGATC |
| *beta-actin* qRT-PCR | |
| qPCR-*actA*-F | TCCTCACCCTCAGATACCC |
| qPCR-*actA*-R | CACCGTCACCAGAGTCCA |
